# Genome-wide identification of *GA2ox* genes family and analysis of *PbrGA2ox1*-mediated enhanced chlorophyll accumulation by promoting chloroplast development in pear

**DOI:** 10.1186/s12870-024-04842-x

**Published:** 2024-03-04

**Authors:** Guoling Guo, Lun Liu, Taijing Shen, Haozhe Wang, Shuqin Zhang, Yu Sun, Guoyu Xiong, Xiaomei Tang, Liwu Zhu, Bing Jia

**Affiliations:** School of Horticulture, Anhui Agricultural University, Hefei, 230036 China

**Keywords:** *PbrGA2ox1*, Chlorophyll accumulation, RNA-seq, Chloroplast development

## Abstract

**Background:**

Chlorophyll (Chl) is an agronomic trait associated with photosynthesis and yield. Gibberellin 2-oxidases (GA2oxs) have previously been shown to be involved in Chl accumulation. However, whether and how the PbrGA2ox proteins (PbrGA2oxs) mediate Chl accumulation in pear (*Pyrus spp.*) is scarce.

**Results:**

Here, we aimed to elucidate the role of the pear *GA2ox* gene family in Chl accumulation and the related underlying mechanisms. We isolated 13 *PbrGA2ox* genes (*PbrGA2oxs*) from the pear database and identified *PbrGA2ox1* as a potential regulator of Chl accumulation. We found that transiently overexpressing *PbrGA2ox1* in chlorotic pear leaves led to Chl accumulation, and *PbrGA2ox1* silencing in normal pear leaves led to Chl degradation, as evident by the regreening and chlorosis phenomenon, respectively. Meanwhile, *PbrGA2ox1*-overexpressing (OE) tobacco plants discernably exhibited Chl built-up, as evidenced by significantly higher *Pn* and *Fv*/*Fm*. In addition, RNA sequencing (RNA-seq), physiological and biochemical investigations revealed an increase in abscisic acid (ABA), methyl jasmonate (MeJA), and salicylic acid (SA) concentrations and signaling pathways; a marked elevation in reducing and soluble sugar contents; and a marginal decline in the starch and sucrose levels in OE plants. Interestingly, *PbrGA2ox1* overexpression did not prominently affect Chl synthesis. However, it indeed facilitated chloroplast development by increasing chloroplast number per cell and compacting the thylakoid granum stacks. These findings might jointly contribute to Chl accumulation in OE plants.

**Conclusion:**

Overall, our results suggested that *GA2oxs* accelerate Chl accumulation by stimulating chloroplast development and proved the potential of *PbrGA2ox1* as a candidate gene for genetically breeding biofortified pear plants with a higher yield.

**Supplementary Information:**

The online version contains supplementary material available at 10.1186/s12870-024-04842-x.

## Introduction

Pear (*Pyrus spp.*) is one of the top three widely cultivated fruit crops globally, with a high nutritional, medicinal, and economic value [1]. The yield and quality of pear are closely related to photosynthesis, which is responsible for carbon fixation, energy supply, and plant growth [2]. The ability of plants to photosynthesize is determined by the level of photosynthetic pigments in the leaves to a certain extent. Of these pigments, chlorophyll, a tetrapyrrole metabolite, is essential for photosynthesis [3, 4]. Thus, fine-tuning Chl is important for enhancing photosynthetic efficiency and increasing fruit crop yield [5]. In other words, plants with greater Chl concentration are of great interest and warrant special attention.

Gibberellin 2-oxidases (GA2oxs) are a distinct class of 2-oxoglutarate-dependent dioxygenase [6]. These enzymes mediate gibberellin (GA) deactivation by irreversibly catalyzing the bioactive GA_9_/_4_ and GA_1_/_20_ or their precursors GA_12_/_53_ into inactive GA_8_/_34_ via 2β-hydroxylation reaction [7]. Due to the crucial functions of GA in plant growth and development [8] and the typical roles of *GA2ox* genes (*GA2oxs*) in GA metabolism, *GA2ox*s have been characterized and widely studied in several plant species, such as *Arabidopsis* [9], tomato [10], rice [11], maize [12], apple [13], and switchgrass [14]. Moreover, extensive research has unveiled the impacts of *GA2oxs* on plant architecture, flowering time, hypocotyl elongation, and seed dormancy in a GA-dependent manner [9, 15, 16].

Notably, apart from the phenotypes of retarded growth, delayed flowering time, and inhibited seed germination [9, 15, 16], *GA2oxs-*overexpressing plants exhibit surprisingly high Chl content. In *Arabidopsis*, *AtGA2ox2* overexpression reportedly facilitates Chl accumulation [17]. Similarly, overexpression of *BnGA2ox2/BnGA2ox6* and *PpGA2ox* in *Arabidopsis* has been shown to significantly increase Chl content [17–19]. In addition, *PcGA2ox1* and *GhGA2ox1* have also been reported to enhance Chl accumulation [20, 21], and transgenic maize and rapeseed plants overexpressing *AtGA2ox1* and *AtGA2ox8* have been stated to display high Chl concentrations [22–24]. These studies highlight the role of *GA2oxs* in Chl accumulation. However, relevant studies revealing how *GA2oxs* work are lacking, whereas the essential role of the pear *GA2ox* gene family in Chl accumulation still needs to be elucidated.

With the inexorable advancement in the RNA sequencing (RNA-seq) technique, there has been an enhancement in the mining and analysis of key factors that are responsible for specific plant traits, making it possible to unveil the regulatory mechanisms underlying the variations in plant characteristics [25]. Previously, RNA-seq helped to reveal the fundamental determinants that contribute to interstock-induced dwarfism in sweet persimmon [26], and altitude-dependent potato tuber coloring [27]. Moreover, RNA-seq has also been widely utilized to derive novel insights into the functions of several genes, such as the *SlWRKY35*-activated carotenoid biosynthesis in tomatoes [28], *MdMYB94*-induced ester biosynthesis in apples [29], and *TgbHLH95-* and *TgbZIP44*-targeted terpene biosynthesis in ‘xiang fei’ (*Torreya grandis*) nuts [30]. Thus, RNA-seq can help efficiently explore the potential mechanisms underlying phenotypic alterations arising from gain-of-function or loss-of-function of genes.

Here, to reveal the role of *PbrGA2oxs* (*GA2oxs* from *Pyrus spp.*) in Chl accumulation and to identify the underlying functional mechanism, the members of *PbrGA2oxs* in pear were isolated using TBtools. We investigated the role of *PbrGA2ox1* in Chl accumulation by transiently overexpressing *PbrGA2ox1* in chlorotic pear leaves, transiently silencing *PbrGA2ox1* in normal leaves, and stably overexpressing it in tobacco plants. Subsequently, we dissected the intrinsic regulatory patterns underlying *PbrGA2ox1*-induced Chl accumulation using transcriptomic analysis on *PbrGA2ox1*-overexpressing (OE) and wild-type (WT) tobacco plants. Fortunately, we identified several key pathways that might contribute to Chl accumulation in OE plants. The role of these pathways was further confirmed by measuring relevant physiological indexes. Furthermore, we explored and verified the effect of *PbrGA2ox1* on Chl synthesis and chloroplast development via transmission electron microscopy. Overall, this study systematically illustrates the role of *PbrGA2ox1* in Chl accumulation, which might broaden our understanding of *GA2oxs*-facilitated Chl accumulation and offer primary insights into the underlying regulatory mechanism.

## Results

### Genome‑wide identification of pear GA2ox genes family and screening of *PbrGA2ox1*

**Fig. 1:**
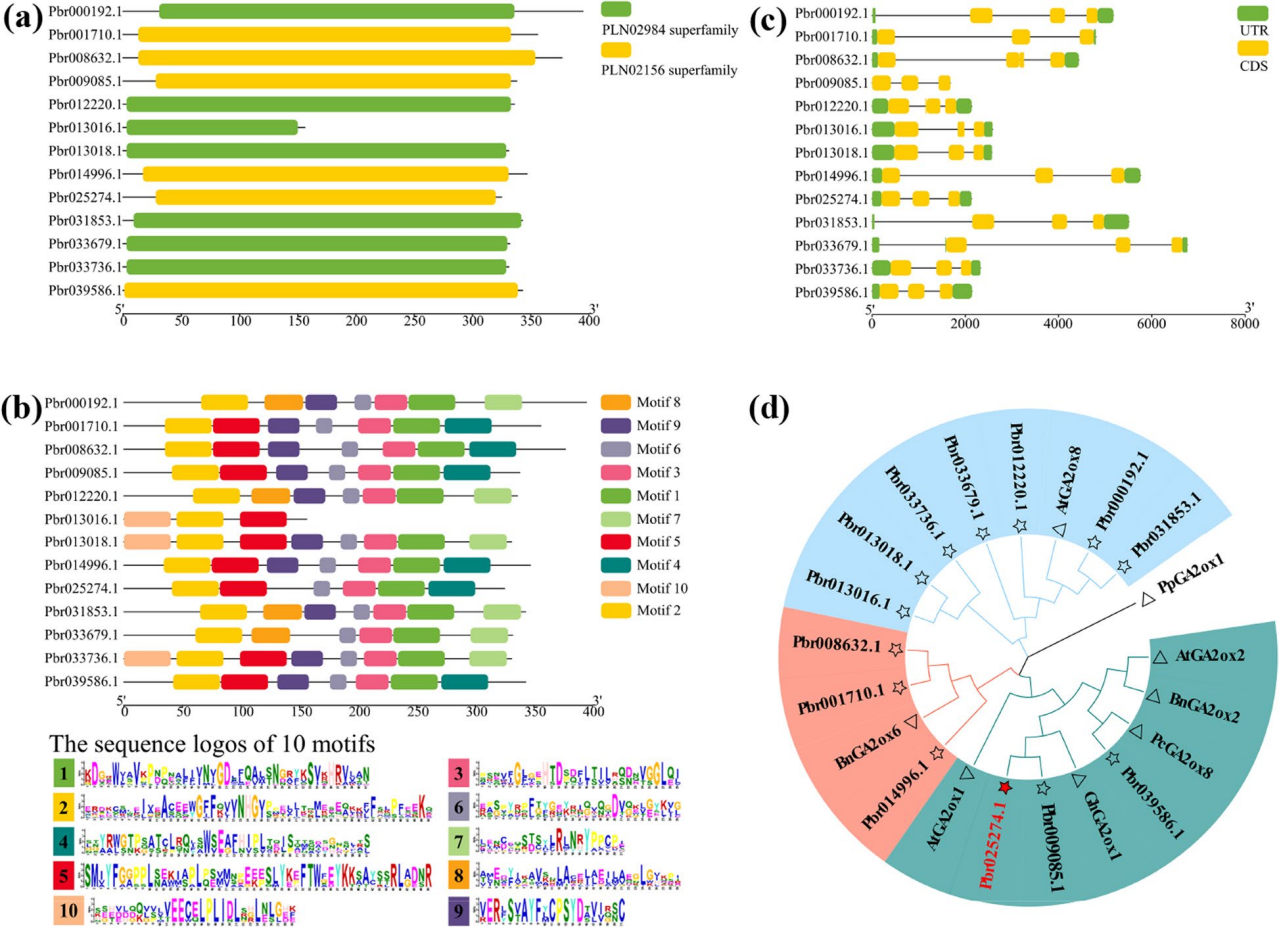
Identification and bioinformatics analysis of pear *GA2ox*-family genes. **a** Conserved domain and (**b**) conserved motifs analysis of pear GA2ox proteins. Different domains and motifs are displayed in different colored boxes. **c** Structure analysis of pear *GA2ox* genes. The exons (coding sequences, CDS) and introns of the genes are marked with yellow boxes and black solid lines, respectively. The untranslated regions (UTR) located upstream or downstream of the genes are indicated by green boxes. **d** Neighbor-likelihood (NL) phylogenetic tree of 13 PbrGA2ox proteins and seven GA2ox proteins involved in chlorophyll accumulation, including three proteins from Arabidopsis (AtGA2ox1, accession number: AT1G78440.1; AtGA2ox2, accession number: AT1G30040.1; AtGA2ox8, accession number: AT4G21200.1), one protein from flowering bean (PcGA2ox8, accession number: AJ132438), one protein from cotton (GhGA2ox1, accession number: XP_016703412), and two proteins from rapeseed (BnGA2ox2, accession number: XP_013671430; BnGA2ox6, accession number: NP_001302845). The tree was constructed in the MEGA 7.0 program with 1000 bootstrap replications

Protein searches of the pear database revealed a total of 13 PbrGA2oxs with lengths ranging from 156 to 395 amino acids (Fig. 1a). These proteins were clustered into three phylogenetic group types (Fig. S1a in Additional File 1) based on the classification of AtGA2ox1; Class I, Class II, and Class III [24]. The proteins harbored only one relatively longer conserved PLN02984 or PLN02156 domain (Fig. 1a). There were ten motifs in PbrGA2oxs, with three to eight motifs randomly distributed in each of these proteins (Fig. 1b). Only one conserved motif was shared by all proteins. The corresponding genes for these 13 PbrGA2oxs possessed at least two introns between the coding sequences (Fig. 1c). and most of them exhibited longer introns, such as *Pbr000192.1* and *Pbr033679.1* (Fig. 1c). These findings showed a diversity in the gene structure of *PbrGA2oxs*. In general, *PbrGA2oxs* in the same class had similar exon–intron structures (Fig. 1c), particularly in terms of number of exons, suggesting that the gene sequence and exon–intron structures are highly conserved within *PbrGA2oxs* of the same class.

To identify the *PbrGA2oxs* potentially involved in Chl accumulation, we performed a phylogenetic tree analysis with GA2oxs that are known to be involved in Chl accumulation (Table S1 in Additional File 2) [17–24]. The three *PbrGA2oxs*, *Pbr025274.1*, *Pbr039586.1*, and *Pbr009085.1*, clustered close to the majority of the other functional GA2oxs (Fig. 1d), indicating that these three genes might contribute to Chl accumulation. Of these three, *Pbr025274.1* was selected for further analyses owing to its higher expression in normal pear leaves than the other two genes (Fig. S1b in Additional File 1). *Pbr025274.1* is annotated as a pear gibberellin 2-beta-dioxygenase 1-like gene in the National Center for Biotechnology Information (NCBI) database, and it was highly homologous to *GhGA2ox1*. Thus, *Pbr025274.1* was designated as *PbrGA2ox1* in this study.

### Functional analysis of *PbrGA2ox1* in Chl accumulation in pear plants

**Fig. 2:**
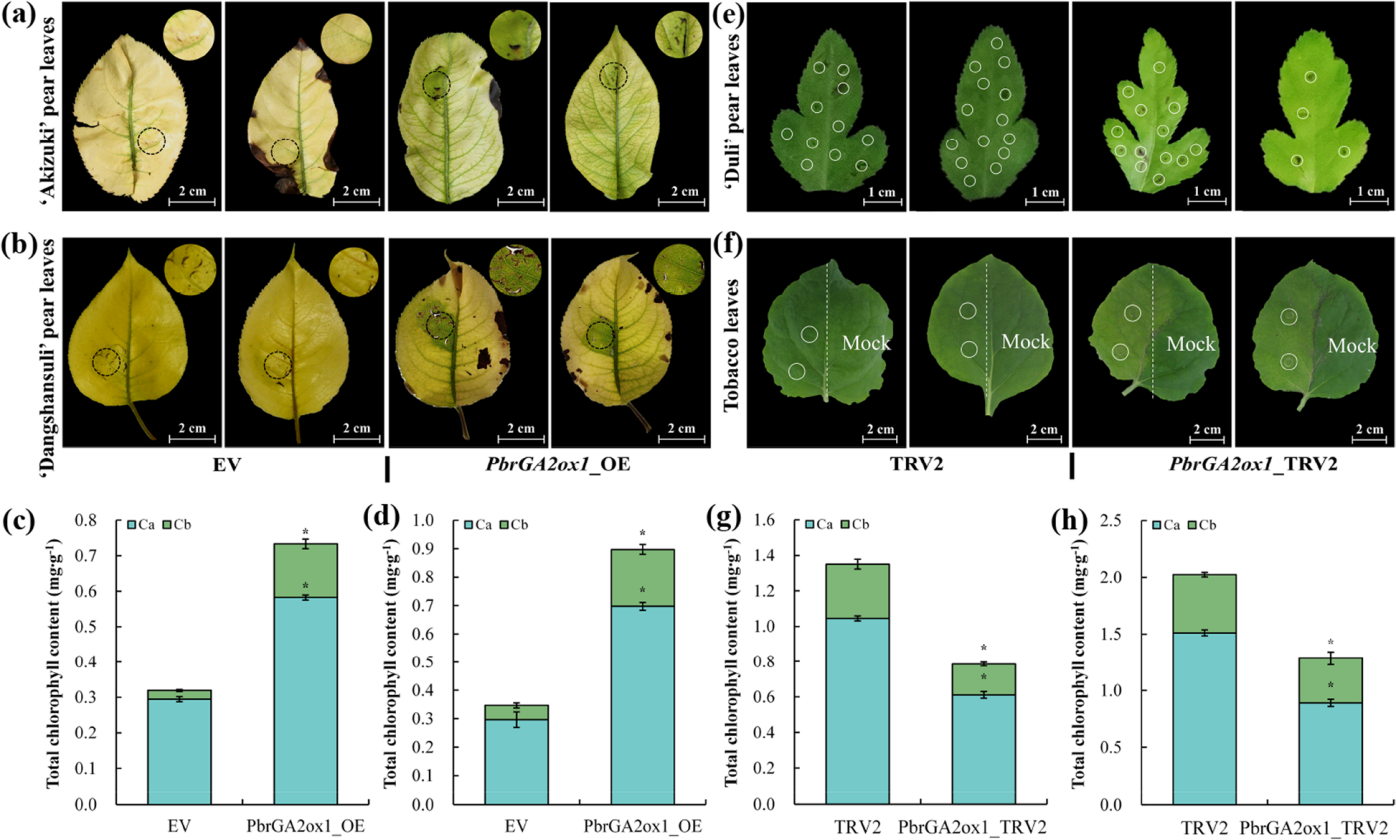
Transient *PbrGA2ox1* overexpression leads to a regreening phenomenon in the chlorotic pear leaf, while its silencing results in the chlorosis of the normal pear leaves. **a**, **c** The outward appearances, and (**b**, **d**) the chlorophyll content of chlorotic leaves from ‘Akizuki’ and ‘Dangshansuli’ pear plants at 14 days after injection with either empty vector (EV) or *PbrGA2ox1*-overexpression construct (*PbrGA2ox1*_OE). The area marked with a dotted circle indicates the injected site in each leaf. **e**, **f** Representative images, and **g**, **h** chlorophyll content of green leaves from ‘Dui’ pear plants and tobacco plants at 14 days after injection with wither either control vector (TRV2, left panel) or *PbrGA2ox1*-silenced construct (*PbrGA2ox1*_TRV2, left panel). Here, whole ‘Duli’ pear leaves and only half of the tobacco leaves were injected and examined, and the white solid circle indicated the injection sites

To investigate the role of *PbrGA2ox1* in Chl accumulation, we generated a *PbrGA2ox1* overexpression construct (*PbrGA2ox1*_OE) and transiently transferred it into the chlorotic leaves of ‘Akizuki’ and ‘Dangshansuli’ pear plants. The leaves injected with the empty vector (EV) were used as the control. To ascertain the efficiency of transformation, a qRT-PCR analysis was carried out with the control and *PbrGA2ox1*‐overexpressing pear leaves seven days post-transformation. The results showed that the *PbrGA2ox1* transcript level in the injection sites of overexpressed leaves was higher than that in the sites of the control leaves in both pear types (Fig. S2a and b in Additional File 1), implying the effectiveness of the system. After 14 days, no significant changes appeared at the injection sites on the control leaves. However, a re-greening phenomenon was observed at the injection sites on the overexpressed leaves (Fig. 2a and b). These findings were further supported by a marked increase in Chl leaves in overexpressed leaves at the end time point (Fig. 2c and d).

Meanwhile, to better understand the role of *PbrGA2ox1* in Chl accumulation, we used the virus-induced genes silence (VIGS) technique to introduce *PbrGA2ox1* knockdown (*PbrGA2ox1*_TRV2) construct in both tobacco and ‘Duli’ pear leaves. The leaves injected with the empty TRV2 vector were used as the control. Again, qRT-PCR was used to quantify *PbrGA2ox1* expression in control and *PbrGA2ox1*-silenced pear leaves. *PbrGA2ox1* expression was markedly suppressed in the silenced leaves than in control leaves (Fig. S2c and d in Additional File 1). These *PbrGA2ox1*-silenced plants were subsequently used for functional analysis. Notably, at 14 days after injection, no discernable differences were observed at the injection site on the control leaves (Fig. 2e and f). However, the injection sites on the silenced leaves exhibited typical chlorosis symptoms (Fig. 2e and f). Furthermore, the *PbrGA2ox1*-silenced exhibited significantly lower Chl content than the control leaves (Fig. 2g and h). These results suggest that *PbrGA2ox1* positively regulates Chl accumulation in pears.

### Effects of *PbrGA2ox1* overexpression on Chl built-up in tobacco plants

**Fig. 3:**
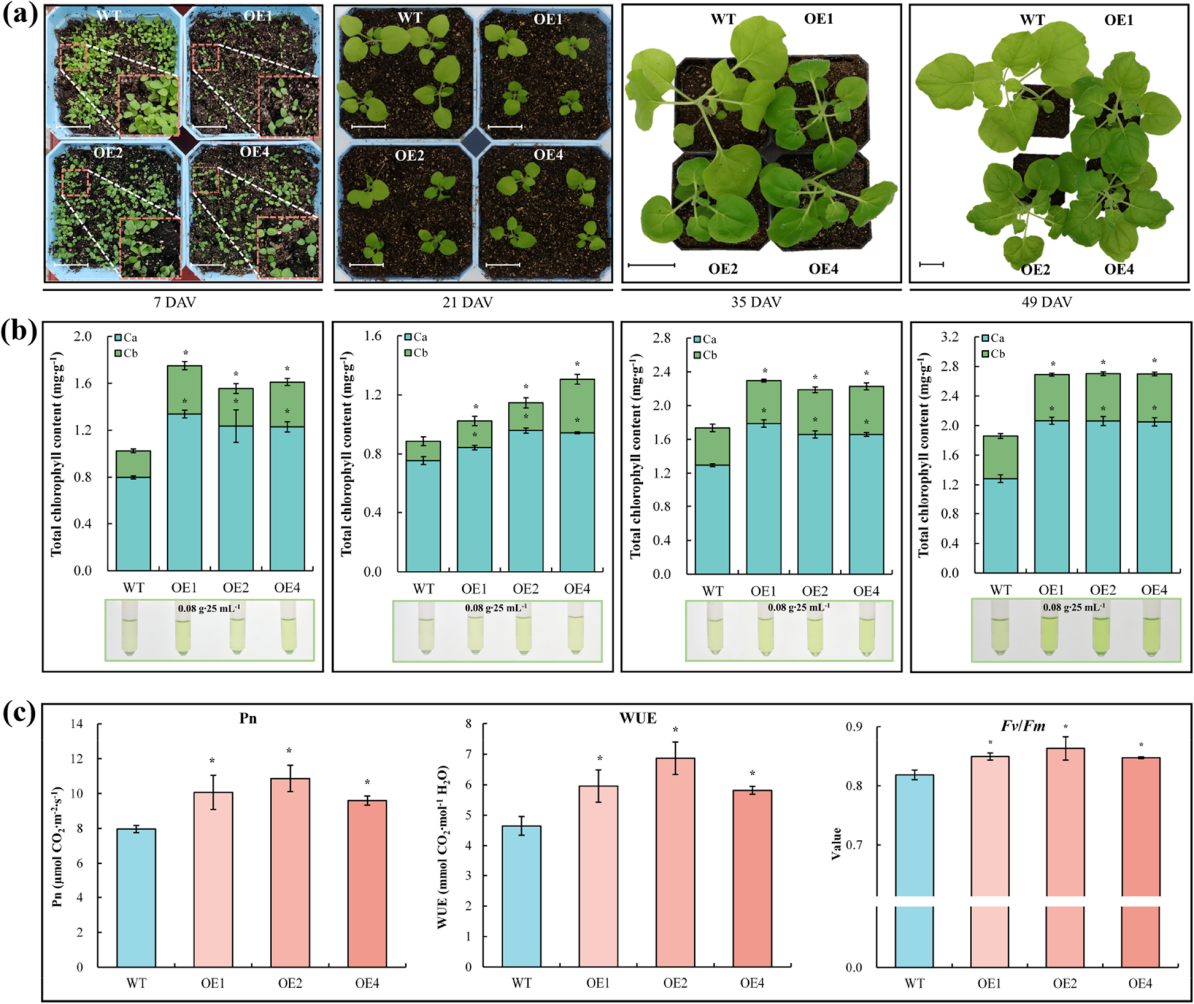
Growth traits and chlorophyll content of wild-type (WT) and *PbrGA2ox1*-overexpressing (OE) tobacco plants under long-day conditions. **a** Phenotypic features of WT and OE transgenic tobacco seedlings from seven days after vernalization (DAV) to 49 DAV. Scale bars = 2.5 cm.** b** Chlorophyll a, chlorophyll b, and the total chlorophyll levels in the leaves of WT and OE transgenic tobacco seedlings at different developmental stages. The data represent the mean ± SD (*n* = 3) of three independent biological experiments. **c** The net photosynthetic rate (Pn), water use efficiency (WUE), and maximal photochemical efficiency of PSII (*Fv*/*Fm*) of WT and OE plants at 35 DAV. The indicator on WT serves as the control. Asterisks used above the columns indicate a significant difference at *p* < 0.05 (one-way ANOVA) to the control based on Tukey’s test

To further validate the physiological impact of *PbrGA2ox1* on Chl accumulation, three previously generated *PbrGA2ox1*_OE tobacco (*Nicotiana benthamiana*) plants (OE_1, OE_2, OE_4) [31] were used. The dynamic growth traits of WT and OE lines were monitored at the seedling stage, with a focus on the leaf color. The results showed that the OE lines exhibited dwarfism but were certainly greener than the WT lines throughout the seedling stages (Fig. 3a). During the later growth stages, Chl a, Chl b, and total Chl levels gradually increased in all plants (Fig. 3a and b). However, their levels in each recorded growth node in the OE plants were prominently higher than in the WT plants (Fig. 3b). Furthermore, the WT and OE lines exhibited discernable variation in the leaf color at 35 DAV (Fig. 3a).

Given the strong association between Chl content and photosynthesis or Chl fluorescence [4, 32–35], the gas exchange and Chl fluorescence parameters were subsequently evaluated. The *Ci*, *Gs*, and *Tr* values did not differ significantly between the OE and WT lines (Fig. S3b, c, and d in Additional File 1). However, we observed markedly higher *Pn* and WUE in the OE plants than in the WT plants (Fig. 3c, Fig S3a and e in Additional File 1). Furthermore, we did not observe significant differences between the *Fm*, *Y*(*II*), *ETR*, *qP, F*_*0*_, *NPQ*, and *Y*(*NPQ*) of WT and OE plants (Fig. S3f, g, i, j, k, l, and m in Additional File 1). However, the *Fv*/*Fm* value of the OE seedlings was markedly higher than that of WT seedlings (Fig. 3c, Fig. S3h in Additional File 1). Altogether, these outcomes suggest that *PbrGA2ox1* overexpression accelerated Chl built-up in tobacco plants.

### RNA-seq and sequencing quality analysis

RNA-seq was used to elucidate the mechanism underlying *PbrGA2ox1* overexpression-mediated Chl accumulation. A total of six cDNA libraries were constructed from tobacco seedlings, including one each from three biological replicates of WT lines (WT_1, WT_2, WT_3) and the three transgenic lines at 35 DAV for transcriptome sequencing. After quality control, an average of 4.87 G of clean reads with GC content ranging from 43.35% to 44.11% was accessed from the RNA-seq data. Around 98% and 94.59% of these six libraries comprised Q20 and Q30 bases, respectively (Table S2 in Additional File 2), indicating that high-quality read sets were obtained. Given the absence of a high-quality reference genome for *N. benthamiana*, the clean reads were mapped to several reported tobacco genomes using TopHat2. An average highest mapping rate of 52.74% was obtained for these clean reads concerning the genome v2.0 of *N. attenuate*. Here, the uniquely mapped clean reads ranged from 50.01% to 51.71% from each library (Table S2 in Additional File 2), highlighting that many uncharacterized or unique genes were still present in *N. benthamiana*. However, owing to the adequate coverage of the RNA-seq data and the high proportions of Q20 and Q30 bases in the raw data (Table S2 in Additional File 2), the transcriptome sequencing data was utilized for subsequent analyses.

### Identification of DEGs

**Fig. 4:**
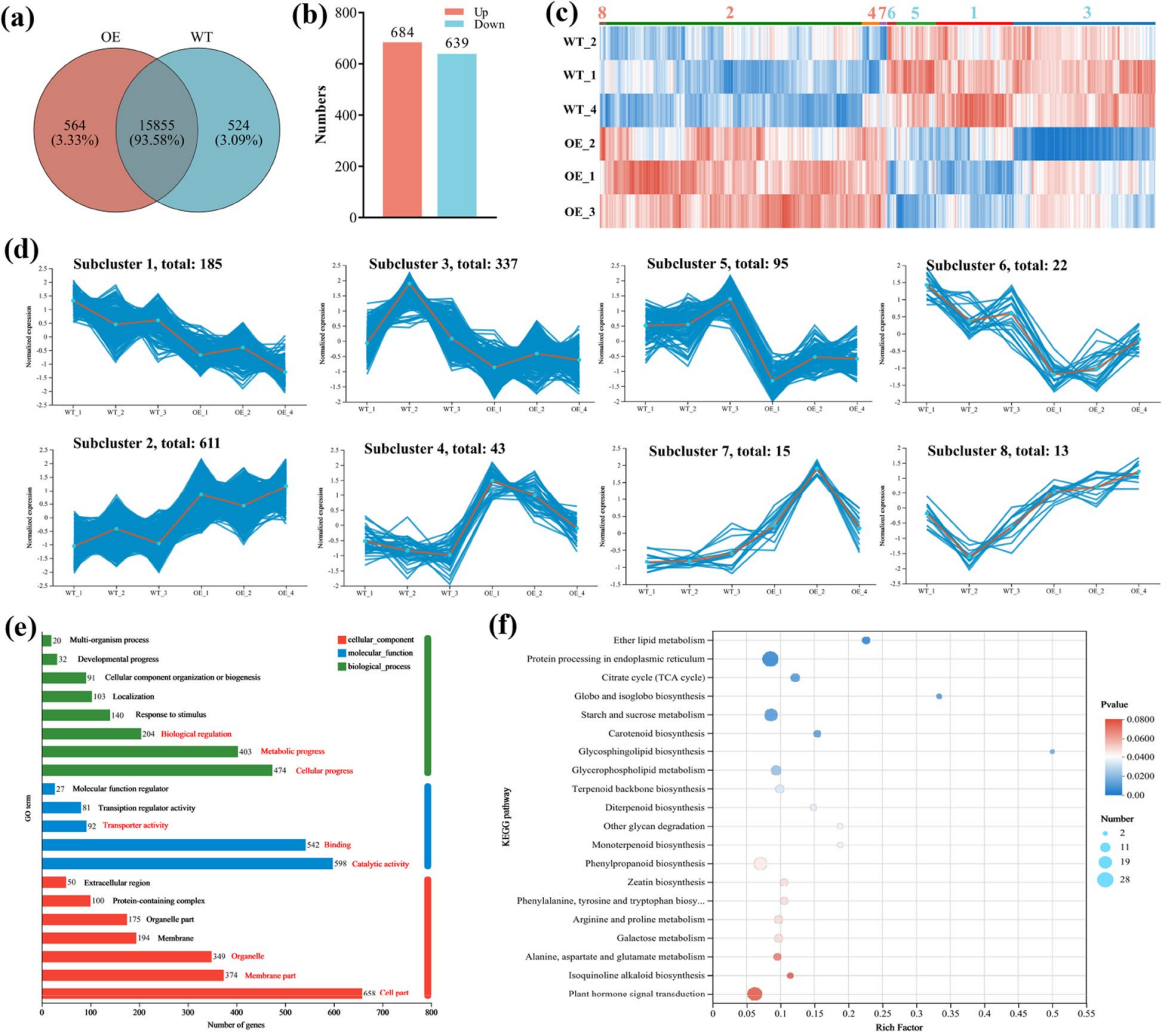
Identification and functional enrichment analysis of differently expressed genes (DEGs). **a** Venn plot analysis of common and uniquely expressed genes in leaves of wild type (WT) and *PbrGA2ox1*-overexpressing (OE) seedlings at 35 days after vernalization (DAV) under long-day conditions. **b** The number of up-regulated and down-regulated DEGs in the comparison group namely OE vs. WT. **c** The expression heatmap of DEGs across total samples. The color indicates the expression level in the form of log_2_|FPKM| values. Blue represents a high expression level, and red represents a low expression level. Numbers above the groups indicate corresponding subclusters from one to eight. **d** Expression profile clustering of the DEGs in OE vs. WT. The red line indicates the average expression level of all DEGs in a subcluster. **e** Enriched gene ontology (GO) terms for DEGs identified from the RNA-seq data of the leaves from WT and OE seedlings. The DEGs were classified based on GO annotation and were categorized into biological process (BP), molecular function (MF), and cellular component (CC). The values indicate the number of genes in each classification, and the red marks highlight the most enriched category. **f** A bubble map of the top 20 KEGG enrichments for DEGs. The ordinate denotes the pathway name, the abscissa describes the richness factor, each circle in the map represents a pathway, the size of a circle indicates the number of DEGs in the pathway, and the color of a circle corresponds to different *p*-value ranges

The contigs were assembled into CDSs of 45899 unigenes with lengths ranging from 200 to 1800 bp, and only 16943 functional genes were annotated in the databases. The Venn diagram showed that 564 and 524 genes were specifically characterized in the OE and WT lines, respectively (Fig. 4a). Thus, 15855 annotated functional genes overlapped in both lines (Fig. 4a). Among the shared genes, the fragments per kilobase of transcript per million mapped reads (FPKM) value were analyzed to further identify the DEGs that might be involved in the Chl deposition in the leaves of OE lines. Thus, 1323 DEGs were identified using DESeq2, with 684 and 639 genes significantly upregulating and downregulating in the OE lines, respectively (Fig. 4b and c). These DEGs were grouped into eight subclusters based on their expression patterns (Fig. 4c and d). Among them, the DEGs from subclusters 2, 4, 7, and 8 were prominently upregulated, and those from subclusters1, 3, 5, and 6 were markedly downregulated in the OE lines compared to the WT lines (Fig. 4c and d). We then analyzed the top 10 upregulated genes with FPKM ≥ 1 from subclusters 2, 4, 7, and 8, and found that they were primarily related to plant responses to abiotic stresses. One of these genes was *NIATv7_g23299*, a member of the P450 family genes [36], which might be involved in regulating chloroplast function (Table S3 in Additional File 2). These results indicate the potential involvement of *PbrGA2ox1* in regulating chloroplast function and abiotic stress responses.

### Functional annotation of DEGs

To identify the *PbrGA2ox1*-regulated biological processes, GO term enrichment was conducted. The GO terms analysis revealed that the DEGs associated with *PbrGA2ox1* overexpression were categorized into the biological process (BP), molecular function (MF), and cellular component (CC) (Fig. 4e). The enriched terms in BP include cellular process, metabolic progression, and biological regulation; those in MF include catalytic activity, binding, and transporter activity; and those in CC include cell part, membrane part, and organelle (Fig. 4e). Consistently, our analysis showed that the selected DEGs were relevant to molecular function (GO:0003674), catalytic activity (GO:0003824), lipid metabolic/biosynthetic process (GO:0006629 and GO:0008610), and DNA-binding transcription factor activity (GO:0003700) (Fig. S4a in Additional File 1). The majority of these genes were significantly upregulated in the OE lines compared to the WT lines (Fig. S5a, b, c, and e in Additional File 2). Therefore, these results indicate enhanced biocatalytic and transcriptional activities in the OE plants.

**Fig. 5:**
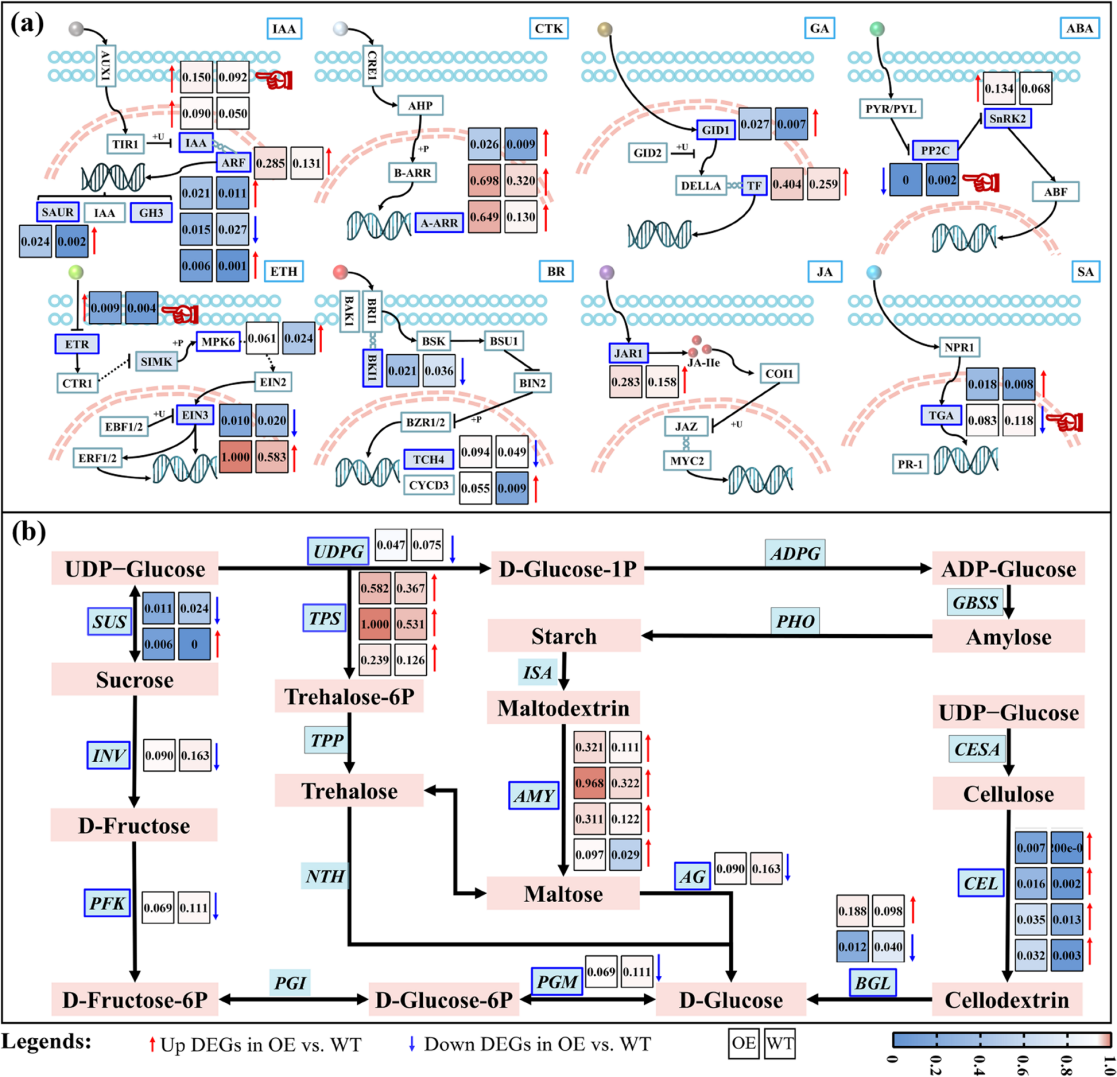
Pathway analysis of differently expressed genes (DEGs). Schematic representation and expression heatmap of DEGs associated with (**a**) hormone signaling, and (**b**) starch and sucrose metabolism. The diagrams were constructed based on the corresponding Kyoto Encyclopedia of Genes and Genomes (KEGG) pathway and literary references. In (**a**), the neatly arranged cyan circles make up the cell membrane and the orange dotted lines comprise the nucleus. In (**a**) and (**b**), the function-annotated DEGs, filtered from the RNA sequencing (RNA-seq) data of the leaves from wild-type (WT) and *PbrGA2ox1*-overexpressing (OE) tobacco seedlings at 35 days after vernalization (DAV) under long-day conditions, are marked with blue boxes. The expression levels for DEGs are shown as min–max normalized counts based on the fragments per kilobase of transcript per million mapped reads (FPKM) values and are presented in colors. The numbers in the boxes are the normalized values, and the blue-to-red gradient denotes a gradual increase in gene expression. The blue arrows indicate a significant decline, whereas the red arrows denote a marked rise in OE vs. WT

Next, we used the KEGG pathway enrichment to gain a broader view of the *PbrGA2ox1*-regulated biological functions. The results revealed that the relevant regulatory pathways related to the DEGs generated by *PbrGA2ox1* overexpression were mainly assigned to metabolism, genetic information and processing, and environmental information processing (Fig. S4b in Additional File 1). These pathways comprise signal transduction, carbohydrate metabolism, terpenoid and polyketide metabolism, lipid metabolism, and amino acid metabolism (Fig. S4b in Additional File 1). Consistently, our analysis showed that the DEGs were enriched in plant hormone signal transduction (map04075), phenylpropanoid biosynthesis (map00940), starch and sucrose metabolism (map00500), and protein processing (map04141) (Fig. 4f). The map04075 pathway primarily comprised the genes related to indole-3-acetic acid (IAA) and ethylene (ETH) signal transduction, the genes related to brassinosteroid (BR) and cytokinin (CTK) signaling (Fig. 5a). In the IAA and ETH signaling, a growth-suppressible gene *NIATv7_g02862* (which is homologous to *AtIAA20*) [37] and a chlorophyll-related gene *NIATv7_g11268* (which is homologous to *MsETR2*) [38] was remarkedly upregulated in the OE lines than in the WT lines (Fig. 5a). This finding implies inhibited growth and increased Chl levels in OE plants.

Moreover, compared to WT plants, three type-A Arabidopsis (*Arabidopsis thaliana*) response regulator (*A*-*ARR*) genes (involved in CTK signaling) and one touch-induced (*TCH*) gene (involved in) in BR signaling were notably upregulated, and one botrytis-induced kinase 1 (*BIK1*) and one *TCH* gene were prominently downregulated in the OE plants (Fig. 5a). Furthermore, seven DEGs, including two TGACG-binding (*TGA*) genes, one phytochrome-interacting factor 3 (*PIF3*), one gibberellin insensitive dwarf 1 (*GID1*) gene, one protein phosphatase 2C (*PP2C*) gene, one SNF1-related protein kinase 2 (*SnRK2*) gene, and one jasmonate response (*JAR*) gene, were involved in the GA, ABA, JA, and SA signal transduction pathways (Fig. 5a). Except for the PP2C gene and one TGA (*NIATv7_g14145*) gene, these genes were upregulated following *PbrGA2ox1* overexpression (Fig. 5a). This outcome indicates that the ABA, SA, and JA signaling pathways were enhanced in the OE lines. Furthermore, we observed a marked elevation in the expression of the genes involved in the conversion of maltodextrin into maltose, UDP-glucose to intrehalose-6P, and cellulose to cellodextrin, including *NbTPS*, *NbAMY*, and *NbCEL*. This finding showed enhanced starch and sucrose metabolisms in the OE lines (Fig. 5b). Taken together, these results indicate the crucial roles of sugar and starch metabolism and hormone signaling in the regulating Chl accumulation of OE lines.

### Expression analysis of DEGs related to Chl synthesis and chloroplast development

**Fig. 6:**
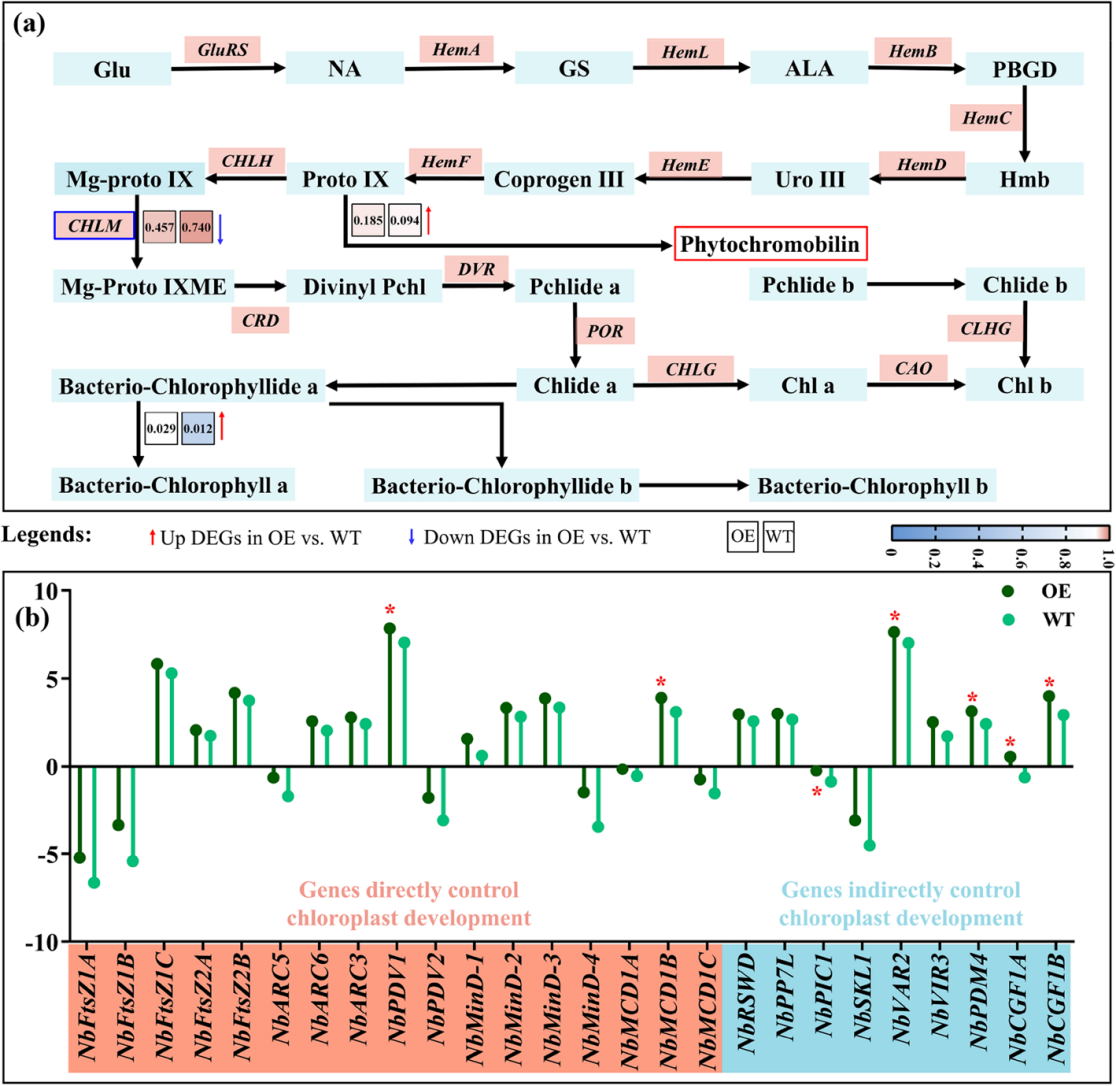
Expression profiles analysis of differently expressed genes (DEGs) involved in chlorophyll synthesis and chloroplast development. **a** Schematic representation and expression heatmap of DEGs related to chlorophyll synthesis in tobacco plants. The diagrams were constructed based on the corresponding Kyoto Encyclopedia of Genes and Genomes (KEGG) pathway and literary references. The function-annotated DEGs, filtered from the RNA sequencing (RNA-seq) data of the leaves from wild-type (WT) and *PbrGA2ox1*-overexpressing (OE) tobacco seedlings at 35 days after vernalization (DAV) under long-day conditions, were marked with blue boxes. The expression levels for DEGs are shown as min–max normalized counts based on the fragments per kilobase of transcript per million mapped reads (FPKM) values and are presented in different colors. The numbers in the boxes are the normalized values, and the blue-to-red gradient denotes a gradual increase in gene expression. The blue arrows indicate a significant decline, whereas the red arrows denote a marked rise in OE vs. WT. Glu: glutamyl; NA: L-glutamyl-tRNA; GS: L-glutamyl-semialdehyde; ALA: 5-aminolevulimate; PBGD: porphobilinogen; Hmb: hydroxymethyl-bilane; Uro III: uropor-phynnogen III; Coprogen III: copropor phyrinogen III; Proto IX: proto IX; Mg-proto IX: Mg-protoporphyrin IX; Divinyl Pchl: divinyl-proto-chlorophyllide; Pchlide a: divinyl-proto-chlorophyllide a; Chlide a: chlorophyllide a; Chl a: chlorophyll a; Pchlide b: divinyl-proto-chlorophyllide b; Chlide b: chlorophyllide b; Chl b: chlorophyll b. **b** Dot chart of the expression level of DEGs directly (orange box) or indirectly (cyan box) regulating chloroplast development in plants. Values are presented in the form of log_2_ based on their FPKM obtained by RNA-seq, the values in WT serve as the control. Asterisks used above the columns indicate a significant difference at *p* < 0.05 (one-way ANOVA) to the control based on Tukey’s test

The emphasis of this work was to investigate the intrinsic mechanisms responsible for the markedly higher Chl content in leaves of OE plants compared to the leaves of WT plants, we analyzed, in-depth, the DEGs involved in Chl synthesis (map00860) (Fig. 6a). Interestingly, the expressions of most of the genes previously characterized to be responsible for the Chl synthesis in tobacco [39, 40] did not differ significantly in OE and WT plants. Based on the RNA-seq data, only three Chl synthesis-related DEGs were isolated in the current study (Fig. 6a). Among these, two DEGs, responsible for the formation of phytochromobilin and bacterio-chlorophyll a, were markedly upregulated in the OE plants than the WT plants (Fig. 6a). In contrast, the third DEG, which is involved in the conversion of protoporphyrin IX to Mg-protoporphyrin IX, was downregulated in the OE lines (Fig. 6a). Meanwhile, we noted that little DEGs existed in the Chl degradation pathway by checking the detail (Data not show). These data suggest that Chl accumulation in the OE plants was not significantly associated with Chl synthesis, as well as Chl degradation, and warrant further investigation to elucidate the primary reason behind Chl accumulation in the OE plants.

Chloroplasts serve as the essential organelles that facilitate Chl synthesis. The number, size, and morphology of chloroplasts directly correlate with the Chl concentration in plants [41]. Since *PbrGA2ox1* overexpression only mildly impacted the expression of Chl synthesis-related DEGs, we examined if it modulated chloroplast development in tobacco. Since the literature on chloroplast development in tobacco is limited [42], we subsequently analyzed the tobacco genes homologous to functional genes previously reported to be directly and indirectly responsible for chloroplast development in *Arabidopsis* [43–49]. We noticed that most of the selected homologous genes were upregulated in the OE plants compared to the WT plants, with significantly elevated expressions of *NbPDV1*, *NbMCD1B*, *NbPIC1*, *NbVAR2*, *NbPDM4*, and *NbCGF1A*/*1B* (Fig. 6b). These findings demonstrate that *PbrGA2ox1* overexpression might enhance chloroplasts development in the OE plants, which might account for the Chl built-up in.

Notably, we also observed the downregulation of two key anthocyanin-induced genes in the OE lines compared to the WT lines (Fig. S6a and b in Additional File 1), indicating suppressed anthocyanin synthesis in the OE plants, which represented an opposing trend to Chl accumulation [50, 51]. In addition, KEGG pathway enrichment also showed that some DEGs were closely associated with MAPK signaling and plant-pathogen interaction (Fig. S6c and d in Additional File 1), suggesting that *PbrGA2ox1* might also play a functional role in MAPK signal transduction and disease resistance. Intriguingly, several stress-responsive transcription factors were also found to be upregulated in the OE lines compared with WT lines (Fig. S7 in Additional File 1), including the *AtWRKY57* [52] homologous gene *NIATv7_g19088*, the *AtDREB2A* [53] homologous gene *NIATv7_g17425*, the *AtEIN3* [54] homologous gene *NIATv7_g18992*, and the *AtWRKY15* [55] homologous gene *NIATv7_g21131* (Fig. S7 in Additional File 1). This observation also implied that OE lines might exhibit better acclimation to stress.

### Validation of RNA-seq data using qRT-PCR

**Fig. 7:**
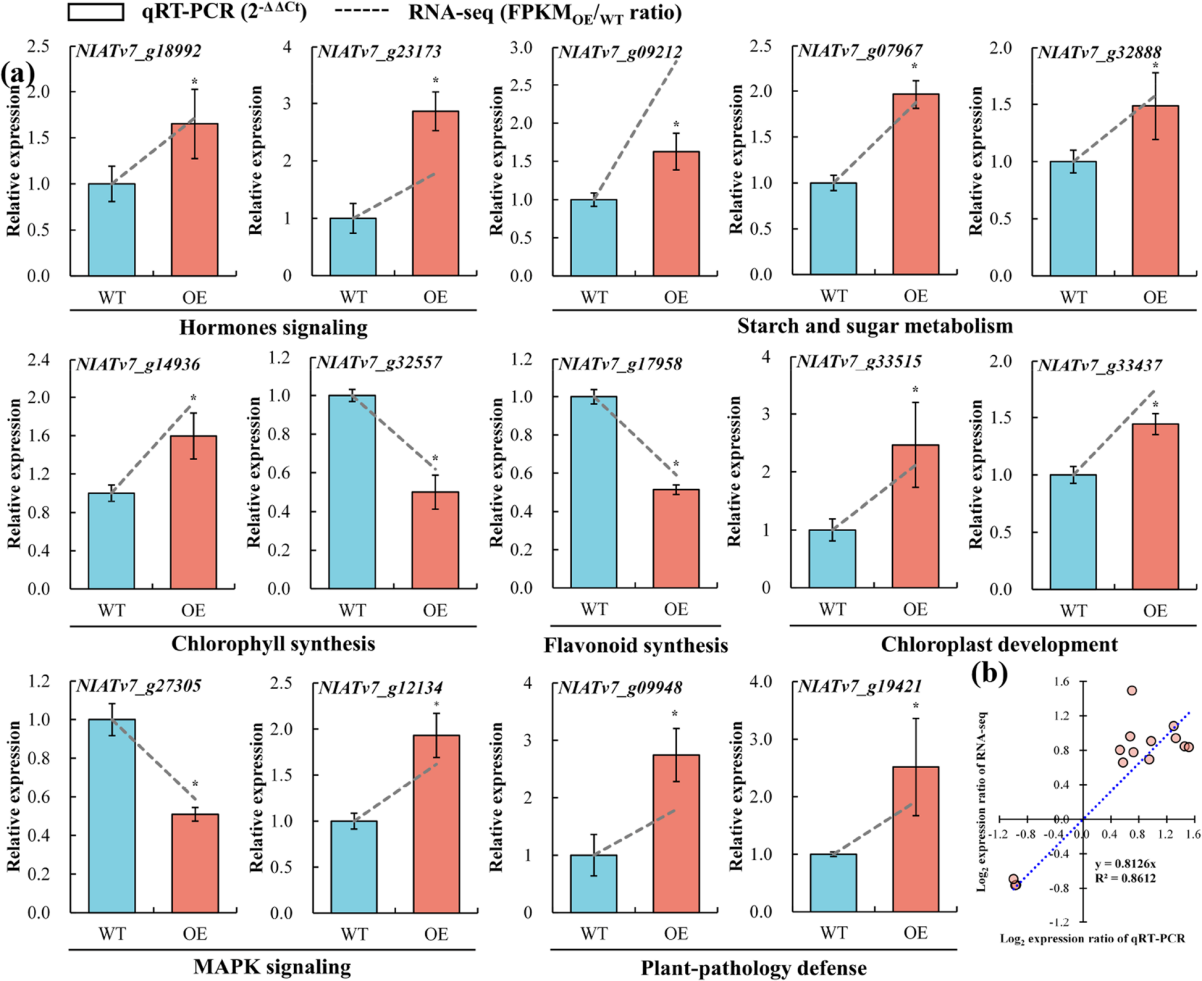
Validation of expression profiles of the 14 selected differently expressed genes (DEGs) by quantitative real-time PCR (qRT-PCR). **a** Relative expression levels of the 14 selected DEGs in leaves of wild-type (WT) and *PbrGA2ox1*-overexpressing (OE) tobacco plants at 35 days after vernalization (DAV) under long-day conditions. The expression levels detected in WT were used as the control and normalized to ‘1’. *NbActin* was used as the internal reference gene. The values represent the mean ± SD of three biological replicates (*n* = 3). Asterisks above the bars indicate a statistically significant difference at *p* < 0.05 (one-way ANOVA) to the control based on Tukey’s test. The dashed trend lines in the bar charts were plotted from the FPKM_OE/WT_ ratio. **b** Pearson correlation scattered plot analysis of the results of qRT-PCR and RNA sequencing (RNA-seq)

To verify the reproducibility of the transcriptomic analysis results, a total of 14 DEGs involved in plant hormone signaling, starch and sucrose metabolism, Chl and flavonoid synthesis, chloroplast development, MAPK signaling, and plant-pathology defense were randomly selected for qRT-PCR confirmation. We observed similar expression patterns for the selected genes in qRT-PCR as was observed during RNA-seq. However, we observed some discrepancies in the fold change of the genes, which might be attributed to the distinct detection ranges and sensitivities of the two analytical methods (Fig. 7a). Moreover, the correlation analysis was conducted using the log_2_ expression ratio of the qRT-PCR-based 2^−ΔΔCt^ values and the log_2_ expression ratio of corresponding RNA-seq-derived FPKM_OE/WT_ values to further validate the reliability of the transcriptomic data. Again, the expression profiles of all the selected genes detected using qRT-PCR were consistent with the RNA-seq results, with the Pearson coefficients (R^2^) value of 0.8612 (Fig. 7b), indicating the high reliability and accuracy of the transcriptomic data.

### Changes in the levels of phytohormones, starch, and sugars in transgenic tobacco plants

**Fig. 8:**
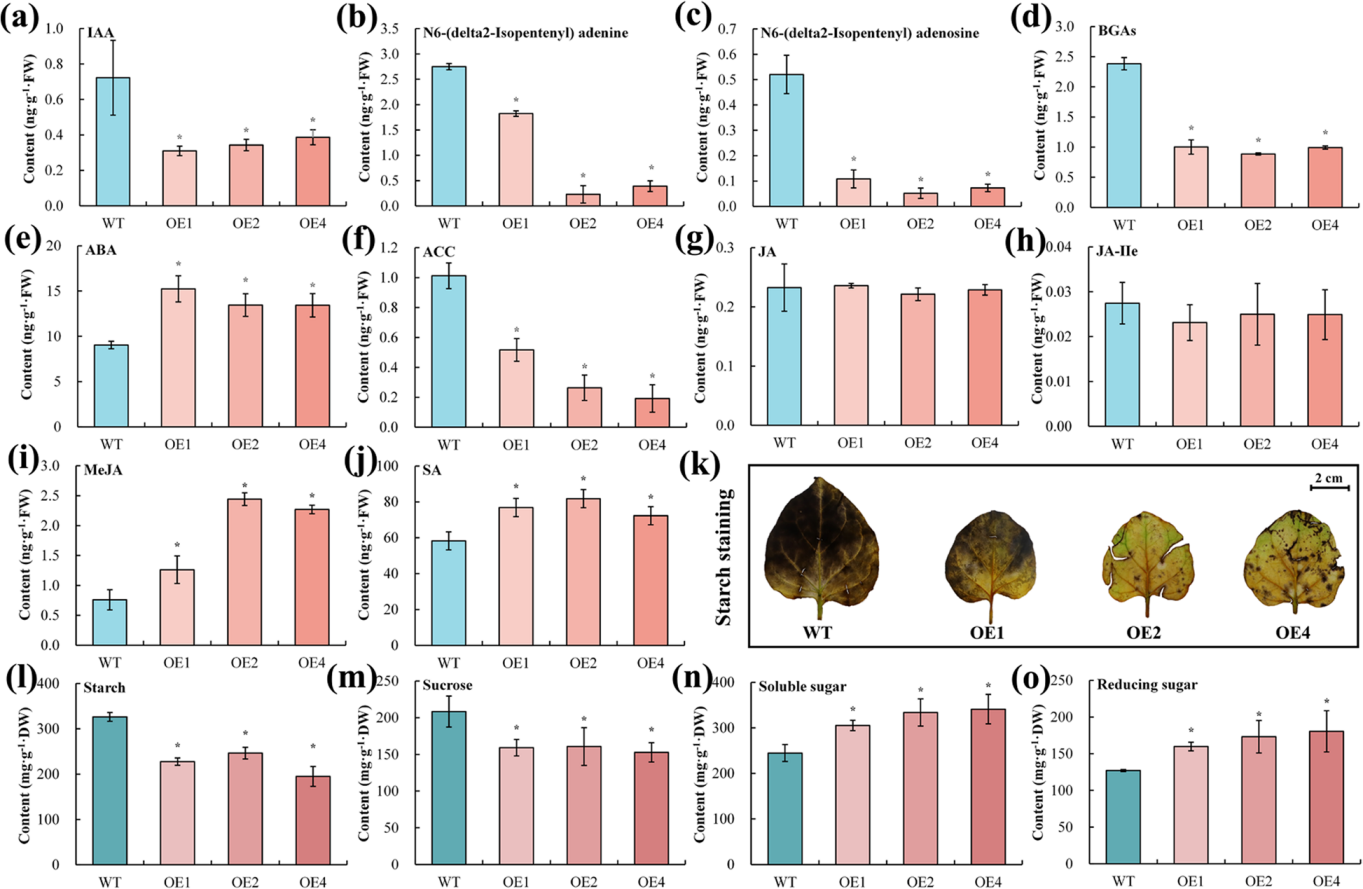
Differences in the levels of hormones and starch and sugar in leaves of wild-type (WT) and *PbrGA2ox1*-overexpressing (OE) tobacco seedlings at 35 days after vernalization (DAV) under long-day conditions. **a-j** The concentration of hormones in the leaves of WT and OE tobacco seedlings at 35 DAV under long-day conditions. The hormones included (**a**) indoleacetic acid (IAA), cytokinin (**b**) N6-(delta2-Isopentenyl) adenine and (**c**) N6-(delta2-Isopentenyl) adenosine, **d** bioactive gibberellins (BGAs), **e** abscisic acid (ABA), **f** ethylene precursor 1-aminocyclopropyl-1-carboxylic acid (ACC), **g** jasmonic acid (JA), **h** jasmonic acid-isoleucine (JA-Ile), (**i**) methyl jasmonate (MeJA) and **j** salicylic acid (SA). **k-o** The abundance of starch and sugar in the leaves of WT and OE tobacco seedlings at 35 DAV under long-day conditions. **k** Starch visualization of WT and OE leaves. **l** Starch content, **m** sucrose content, **n** soluble sugar content, and **o** reducing sugar content in WT and OE leaves. Data are shown as the mean ± SD (*n* = 3) of three independent biological experiments. The indicator of WT serves as the control, ‘ns’ and asterisks used above the columns indicate insignificant and significant differences at *p* < 0.05 (one-way ANOVA) to the control based on Tukey’s test, respectively

Subsequently, we investigated the levels of some phytohormones, sugar, and starch in the leaves of WT and OE plants at 35 DAV. The results showed that the OE plants exhibited extremely lower IAA, bioactive gibberellins (BGAs, GA_1+3+4+7_), and 1-aminocyclopropane-1-carboxylic acid (ACC) (Fig. 8a, d, and f) compared to those of WT plants, whereas the contents of JA and JA-Ile did not differ significantly between OE and WT plants (Fig. 8g and h). In addition, a similar downward trend was observed for N6-(delta2-Isopentenyl) adenine and N6-(delta2-Isopentenyl) adenosine, two kinds of cytokinin (CTK), in OE seedlings (Fig. 8b and c). On the contrary, the OE seedlings possessed pronouncedly higher contents of ABA, MeJA, and SA than the WT seedlings (Fig. 8e, i, and j).

We used histochemical staining to analyze the differences in the starch contents of WT and OE plants. We observed fewer staining spots and less intense staining on the leaves of OE seedlings than WT seedlings (Fig. 8k). Moreover, the quantitative analysis also revealed considerably lower starch and sucrose contents in the OE plants compared to the WT plant (Fig. 8l and m). Consistently, the activity of the β-amylase, a vital enzyme responsible for starch degradation [56], in OE plants was prominently higher, and the expression of its two encoding genes (BAMs) were also remarkably elevated in OE plants compared to those in WT plants (Fig. S8 in Additional File 1). More importantly, notably higher levels of soluble sugar and reducing sugar were detected in the OE lines than in the WT lines (Fig. 8n and o). Overall, these results demonstrated that *PbrGA2ox1* overexpression-mediated Chl built-up was tightly regulated via the accumulation of ABA, JA, MeJA, soluble sugar (except for sucrose), reducing sugar, and the decline of IAA, BGAs, ACC, and CTK, starch, and sucrose.

### Effects of *PbrGA2ox1* overexpression on chloroplast morphology and ultrastructure

**Fig. 9:**
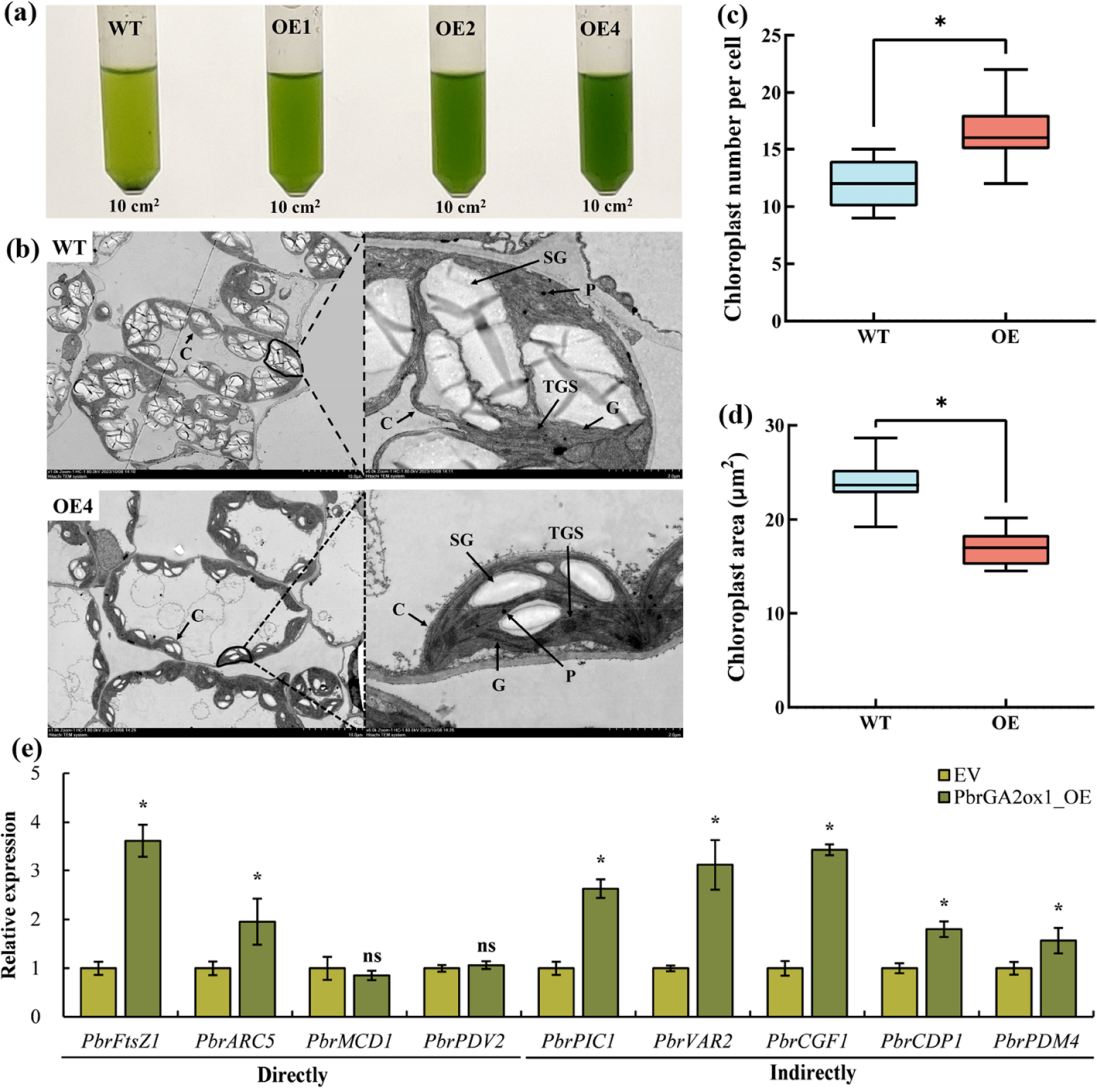
Analysis of chlorophyll development in tobacco and expression profiles of related genes in pear. **a** Visualization of chloroplast abundance in the leaves (10 cm^2^ section) of wild-type (WT) and *PbrGA2ox1*-overexpressing (OE) tobacco seedlings at 35 days after vernalization (DAV) under long-day conditions using the grinding method. **b** The ultrastructure of chloroplasts from the leaves of WT and OE plants. C, chloroplast; S, starch granule; P, plastoglobuli; G, granum; TGS, thylakoid granum stacks. Scale bars: 10 μm (left) and 2 μm (right). **c** Number, and (**d**) size of chloroplasts per cell in the leaves of WT and OE seedlings at 35 DAV. The data represent the mean ± SD of values obtained from at least ten chloroplasts from five individual cells (*n* = 5). **e** Expression analysis of the genes related to chloroplast development in the spots injected with empty vector (EV) or *PbrGA2ox1*-overexpression construct (*PbrGA2ox1*_OE). The gene expression level detected in EV-injected spots was used as the control and set to ‘1’. *PbrActin* was used as the internal reference gene. Values shown are the mean ± SD of three biological replicates (*n* = 3). ‘ns’ and asterisks above the bars indicate insignificant and significant differences at *p* < 0.05 (one-way ANOVA) to the control based on Tukey’s test

Next, we collected chloroplasts from equal areas of the leaves of the WT and OE lines and resuspended the precipitates in a chloroplast storage buffer. We observed that the solution containing the chloroplasts collected from the OE plants, especially for the OE4 plants, was greener than the solution containing the chloroplasts collected from the WT plants. This finding showed that the OE plants contained more chloroplasts than the WT plants (Fig. 9a). Further while analyzing of chloroplast ultrastructure using TEM, we found that while the chloroplasts in the WT plants were uniform, with overtly visible thylakoid granum stacks; however, the chloroplasts in OE4 plants were more strongly connected in a chain and attached more tightly to the cell walls (Fig. 9b).

More importantly, compared to the WT plants, the OE4 plants exhibited denser thylakoid granum stacks, and smaller starch granules (Fig. 9b). Moreover, we discovered an appreciably higher number of chloroplasts per cell in OE4 plants than in the WT plants (Fig. 9b and c). The size of the chloroplasts in OE4 plants was conspicuously smaller than in the WT plants (Fig. 9b and d). However, the cell size in the former was dominantly diminished than the latter (Fig. S9 in Additional File 1), demonstrating more cells per area and consequently, more chloroplasts per area in the OE plants. These observations showed that *PbrGA2ox1* promotes Chl accumulation by facilitating chloroplast development in tobacco.

Therefore, given the crucial role of *PbrGA2ox1* in facilitating chloroplast development, we used TEM to observe the chloroplasts ultrastructure of the infected spots in ‘Dangshansuli’ pear leaves (Fig. 2b), since they displayed a marked regreening phenomenon. Unfortunately, we failed (Fig. S6e in Additional File 1). Thereafter, we turned to focus on the expression of the genes related to chloroplast development in pear plants. Literature is scarce on the pear genes associated with chloroplast development. Hence, we used the BioEdit software to identify the pear genes that were homologous to the aforementioned genes related to chloroplast development in Arabidopsis and tobacco [42–49]. Based on their homology, the identified pear genes were designated as *PbrFtsZ1*, *PbrARC5*, *PbrMCD1*, *PbrPDV2*, *PbrPIC1*, *PbrVAR2*, *PbrCDF1*, *PbrCDP1*, and *PbrPDM4*. Of these, we observed no discernable differences in the expressions of *PbrMCD1* and *PbrPDV2* in control and OE spots, but the remaining genes were markedly upregulated in the latter (Fig. 9e), indicating that the development of chloroplast in the spots was also more or less stimulated. Altogether, these findings presented that *PbrGA2ox-*induced Chl accumulation was primarily dependent on improved chloroplast development, but not the Chl synthesis pathway.

## Discussion

### Discovery of the GA2ox gene family in pear

Chl is a dominant pigment in plants and plays a vital role in photosynthesis [3]. It harvests light and converts it into chemical energy to facilitate the growth and development of plants [57]. Hence, Chl accumulation helps enhance photosynthesis, promote plant growth, and thus improve crop productivity [4]. *GA2oxs* are key players in GA inactivation [7], with critical roles in plant growth and development [58, 59]. Several previous studies have shown that besides regulating seed germination, plant flowering, and morphogenesis [9, 16], GA2oxs also modulate the Chl accumulation in many plants [17, 18, 20, 22, 24]. However, the role of *GA2oxs* in the Chl accumulation of pear is still unknown. Even the pear *GA2oxs* have not yet been identified. Therefore, identifying the pear *GA2ox* members and their role in Chl accumulation is of great significance.

In this study, a total of 13 PbrGA2oxs in pear were isolated and characterized based on their homology with AtGA2oxs (Fig. 1). The isolated PbrGA2oxs were divided into three classes (I, II, and III) based on the phylogenetic analysis. Classes I, II, and III comprised three, three, and seven members, respectively (Fig. S1a in Additional File 1), which followed the previous evolutionary works on GA2oxs [9, 10, 12]. Protein domain analysis revealed that the distribution pattern of the PLN02984 and PLN02156 domains across PbrGA2ox was similar to that of the AtGA2oxs [9] (Fig. 1a), suggesting a deterministic role of these domains in gene function. In addition, motif analysis uncovered the presence of a set of 10 motifs in PbrGA2oxs (Fig. 1b), several of which are identical to those found in GA2oxs of maize [12], rice [11], and Arabidopsis [9], implying a specific function of GA2oxs in plants. However, among the 10 identified motifs, only one motif was common across the 13 PbrGA2oxs (Fig. 1b), indicating functional diversity among PbrGA2oxs. Furthermore, gene structure analyses showed that the average intron length of these 13 *PbrGA2oxs* was longer than their average exon length (Fig. 1c), offering greater flexibility in terms of exon rearrangement, and thereby conferring the genes with unique function, such as the facilitation of Chl accumulation.

### Exploration of *PbrGA2ox1*-driven Chl accumulation

We established a phylogenetic tree of the *PbrGA2ox* gene family with multiple other GA2oxs that are known to have participated in Chl accumulation [17–24]. Of these, *Pbr025274.1*, *Pbr009085.1*, and *Pbr039586.1* exhibited closer relationships with most other GA2oxs, indicating that these might be the key regulators of Chl accumulation in pear (Fig. 1d). Among these three, *Pbr025274.1* exhibited the highest expression level in normal pear leaves (Fig. S1b in Additional File 1). It was renamed as *PbrGA2ox1* and was further analyzed for its role in Chl accumulation via transient overexpression and silencing in chlorotic and normal pear leaves, respectively (Fig. 2).

Callus and root transformation systems are efficient and rapid genetic transformation methods used to study the gene function in pears [60, 61]. Previous studies have used these systems to study the gene function in pears. However, these studies primarily focused on anthocyanin accumulation [62], stone cell formation [63], and abiotic stress responses [64]. Since the application of these methods has not been reported to assess Chl accumulation, we instead used transgenic tobacco plants with *PbrGA2ox1* overexpression for an in-depth analysis of the role of this gene in Chl accumulation. We observed a superior Chl accumulation in the transgenic tobacco plants during dynamic development than in the WT plants (Fig. 3a and b). Furthermore, the OE plants exhibited notably enhanced *Pn* and *Fv*/*Fm* (Fig. 3c, Fig. S2a and h). These findings followed the previous studies stating that plants with the potential for Chl accumulation usually developed a dark green phenotype [65, 66] and typically exhibited better photosynthetic performance [67, 68]. Overall, these results were in line with the observation of transient assay on pear leaves and indicated that *PbrGA2ox1* is involved in Chl accumulation, similar to *AtGA2ox2* and *GhGA2ox1* in *Arabidopsis* [17] and cotton [21].

### Modified pathways in OE tobacco plants

Emerging evidence suggests that *GA2oxs* are important for Chl accumulation [17–19, 21, 22, 24]. However, the underlying mechanisms remain elusive. As a time-saving, sensitive, and efficient technique, transcriptomic analysis has been extensively applied to elucidate the intrinsic mechanisms inherent in the function of several genes [25, 69]. Therefore, to shed light on the regulatory metabolism underlying Chl accumulation mediated by *PbrGA2ox1* overexpression, we constructed six cDNA libraries and employed the RNA-seq technique (Fig. 4, Table S2 in Additional File 2). GO enrichment analysis revealed that *PrbGA2ox1* primarily regulated the genes involved in molecular function, catalytic activity, and lipid metabolic/biosynthetic processes (Fig. 4e, Fig. S4a in Additional File 1). In addition, the KEGG analysis showed that the modified genes were enriched in metabolism, genetic information, processing, and environmental information processing (Fig. 4f, Fig. S4b in Additional File 1). Furthermore, compared to the WT plants, the OE plants exhibited profound alterations in plant hormone signal transduction and starch and sucrose metabolic pathways (Fig. 5), which might be responsible for the dark green color of the leaves of OE plants.

### Altered hormone signaling might account for *PbrGA2ox1*-promoted Chl built-up

Phytohormones are small molecules that modulate diverse physiological and cellular responses and play crucial roles in mediating Chl accumulation [70, 71]. Extensive investigations have revealed the positive roles of IAA, CTK, GA, and SA [72–75], and the negative roles of ETH, JA, and ABA in Chl accumulation [76–78]. However, Previous studies reported a marked decline in the Chl content in oil palm [79] and *Arabidopsis* [80] following GA_3_ and IAA treatments, respectively. On the other hand, some studies reported a marked elevation in the Chl level in Chinese cabbage [81] and citrus [82] after exogenous application of appropriate doses of ABA and MeJA, respectively. Moreover, wheat with markedly reduced active CTK due to the overexpression of *cZOGT1*, a cis-zeatin-O-glucosyltransferase gene, indeed displayed greener phenotypes than non-transformed plants [83]. These findings indicated that the impact of plant hormones on Chl accumulation varies widely based on the plant species and the dosages used.

In the current study, our results support that ABA, SA, and JA facilitated, and IAA, CTK, GA, and ETH inhibited Chl accumulation in plants, which was inferred from the observation of significantly declined contents of IAA, CTK, GA, and ACC, and the prominently escalated abundances of ABA, SA, and MeJA in the OE plants compared to WT plants (Fig. 8 a, b, c, d, e, f, g, h, and i). In agreement with the inference, we also found impaired IAA and ETH signaling, and enhanced ABA, SA, and JA signaling in OE plants compared to WT plants, as evident by the dramatically up-regulated expression of core repressors in IAA (*AUX*/*IAA*) and ETH signaling (*ETR*) and master facilitators in ABA (*SnRK*), JA (TGA), and JA signaling (*JAR1*) (Fig. 5a). Namely, *PbrGA2ox1*-stimulated Chl accumulation was found to be partially associated with hormone metabolism and signaling. Moreover, as plant growth regulators, IAA, GA, and CTK favor plant development, while ABA and JA restrict plant growth [84–86]. Thus, these changes in the levels of IAA, GA, CTK, ABA, and JA in OE plants (Fig. 8a, b, c, d, e, g, h, and i) could well explain the cause of the dwarfism among these plants.

### Increased sugar levels might account for the *PbrGA2ox1*-sponsored Chl accumulation

In addition to the variations in hormonal levels, we also noted a striking reduction in starch and sucrose contents within the OE plants than the WT plants, followed by a pronounced rise in amylase activity and the levels of soluble and reducing sugar content (Fig. 8k, l, m, n, and o, Fig. S8a in Additional File 1). In line with the altered sugar content, we also observed a great upregulation of the genes responsible for starch and sucrose metabolism, such as *NbTPS*, *NbAMY*, *NbCEL*, and *NbBAM* in the OE plants (Fig. 5b, Fig. S8b, c in Additional File 1). These results suggested a significantly intensified conversion from polysaccharides to monosaccharides in the OE plants, which could partly explain the advanced starch and sucrose degradation in the greener OE plants (Fig. 8k, l, and m). For cost reasons, we could not concretely determine the contents of the respective components of the reducing and soluble sugars. However, the main sugars that exist in tobacco are glucose, fructose, and sucrose [87]. It’s well established that glucose and sucrose exhibit dual roles in mediating Chl accumulation in a dose- and species-dependent manner [88–93]. Therefore, alteration in the levels of endogenous sugars might be another key factor contributing to the Chl accumulation in the OE plants. Based on the obtained results, we speculate an accelerating and a suppressive effect of glucose and sucrose in Chl built-up, respectively. Nevertheless, few studies have focused on the effects of fructose on Chl content. Hence, we could not speculate on the relationship between fructose and Chl accumulation. These aspects need to be further investigated.

### *PbrGA2ox1*-stimulated Chl accumulation primarily depends on the enhanced chloroplast development

Chl biosynthesis is an intricate process involving at least 15 enzymes encoded by more than 20 genes [94, 95]. An enhancement or weakening of any of these processes due to the overexpression or silencing of the related genes can considerably affect Chl levels in plants, resulting in yellow − dark green leaves [96, 97]. However, in the present work, we discovered that only three of these Chl biosynthetic genes were differentially expressed between OE and WT plants (Fig. 6a, Fig. S9 in Additional File 1), implying that *PbrGA2ox1* overexpression has limited effects on enhancing the Chl synthesis system. Moreover, ABA/JA/SA- and sugar-induced Chl accumulation was also poorly correlated with the Chl biosynthesis.

The chloroplast is a central site for a myriad of fundamental cellular processes to take place. It is most well recognized for its role in Chl biosynthesis [41, 44]. Previous studies have reported that the Chl content in plants is tightly linked to the chloroplast development in the cells, including its biosynthesis and division (size and number), and integrity (morphology) [98, 99]. These characteristics are regulated by multiple chloroplast-encoded structural genes and nuclear-localized transcriptional genes [43, 44]. Consequently, we speculated that Chl accumulation in OE plants is closely associated with chloroplast development in these plants. Our analyses revealed a significant upregulation of the genes related to chloroplast development in OE plants compared to WT plants, in particular, *NbPDV1*, *NbMCD1B*, *NbPIC1*, *NbPDM4* and *NbCGF1A*/*1B* (Fig. 6b). These results suggested more developed chloroplasts in OE plants than in WT plants. Consistently, compared to WT plants, we observed more chloroplasts per cell in OE plants, and the chloroplasts were better‐organized with denser thylakoids (Fig. 9a, b, and c). Though the chloroplasts in the OE plants were smaller than those in the WT plants (Fig. 9d), the number of chloroplasts per area in OE plants might be greater owing to the diminished cell size in these OE plants (Fig. S9 in Additional File 1). It further provides an opportunity for the OE plants to accumulate more Chl. These data aligned with the previous reports that proposed the positive effects of chloroplast size, number, and integrity on Chl accumulation [100, 101].

We could not analyze, in detail, the chloroplast ultrastructure in pear leaves suspended in the infiltration buffer containing *PbrGA2ox1*-targeted strains. However, these leaf samples exhibited notable upregulation of *PbrFtsZ1*, *PbrARC5*, *PbrPIC1*, *PbrVAR2*, *PbrCDF1*, *PbrCDP1*, and *PbrPDM4* (Fig. 9e). These results are in agreement with the prior documented functions of *PDV*, *MCD*, *VAR*, *PIC1*, *PDM*, and *CGF* in regulating chloroplast development [42, 45–49]. These outcomes further proved that well-developed chloroplasts facilitate Chl accumulation [99, 101, 102]. On the whole, our results revealed a key mechanism underlying enhanced Chl accumulation in OE plants.

Beyond those, hormones and sucrose have been stated to critically influence chloroplast development [43, 103]. For instance, ABA and sucrose have respectively been found to positively and negatively regulate both thylakoid granum stacks and chloroplast differentiation [103, 104], which was in line with the denser thylakoid, dominantly increased ABA content, and strikingly declined sucrose content in the OE plants in the current study (Fig. 8e and m, Fig. 9e). Therefore, we deduced that modified metabolisms of hormones, starch, and sucrose might be involved in chloroplast development. Cumulatively, *PbrGA2ox1* promotes Chl accumulation primarily by promoting chloroplast development, with a focus on the intensification of the thylakoid membrane.

## Conclusion

**Fig. 10:**
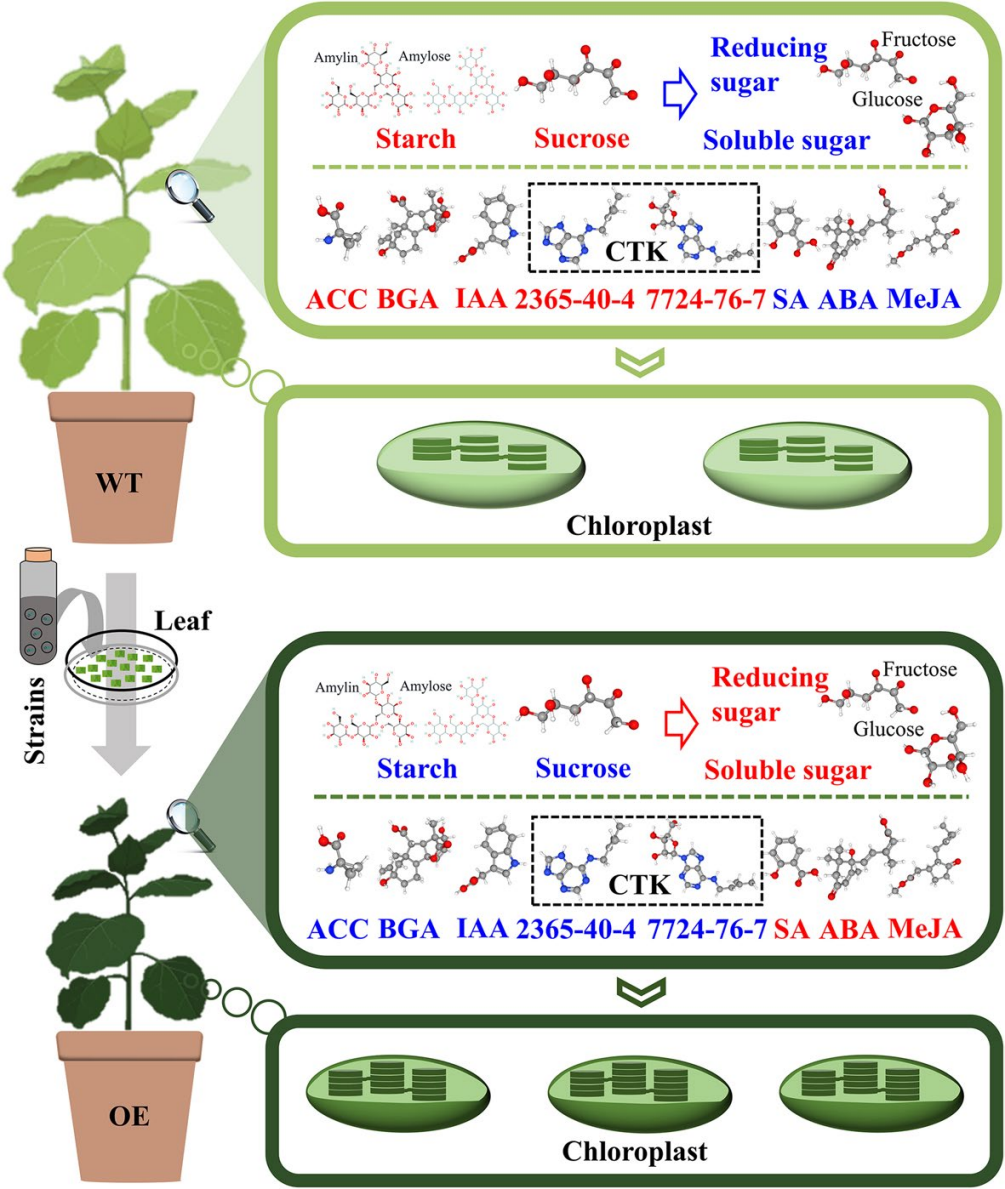
Schematic model showing the potential mechanism underlying *PbrGA2ox1*-induced chlorophyll accumulation in tobacco plants. WT, wild-type tobacco plants; OE, *PbrGA2ox1*-overexpresing tobacco plants; ACC, 1-aminocyclopropyl-1-carboxylic acid; SA, salicylic acid; ABA, abscisic acid; BGA, bioactive gibberellin; IAA, indoleacetic acid; MeJA, methyl jasmonate; 2365–40-4, N6-(delta2-isopentenyl) adenine; 7724–76-7, N6-(delta2-isopentenyl) adenosine. The red font and red arrows indicate a higher level and a faster conversion process, respectively, whereas the blue font and blue arrows imply a lower level and a slower conversion process, respectively. The tobacco model was accessed from the BioRender online website (https://www.biorender.com/)

In summary, a total of 13 *PbrGA2oxs* belonging to three subclusters were isolated and characterized from the pear database. Of these, *PbrGA2ox1* was identified as a positive regulator of pear Chl accumulation. It was found to alter the composition of hormones and saccharides and modulate the expression of genes involved in chloroplast development, thereby mediating the relevant signaling pathways and comprehensively stimulating the proliferation and intensification of chloroplasts. This, in turn, typically provides more and better sites for Chl synthesis, ultimately resulting in enhanced Chl accumulation (Fig. 10). Collectively, our results show that *PbrGA2ox1* contributes to Chl accumulation and provides evidence for enhanced chloroplast development as the core pathway underlying *GA2ox*s-promoted Chl accumulation in plants.

## Materials and methods

### Plant materials and growth conditions

All experiments were carried out with the permission of the relevant agencies or farmers in charge. Chlorotic and normal pear leaves use were obtained from four-year-old Japanese pear ‘Akizuki’ (*Pyrus pyrifolia* Nakai) plants in the open field of the Xiaoxian County, Anhui Province, China, and 55-year-old local ‘Dangshansuli’ (*P. bretschneideri* Rehd.) plants grafted onto ‘Duli’ (*P. betulaefolia* Bunge.) tree rootstocks in the open field of the Dangshan County, Anhui Province, China, respectively. The chlorosis of pear plants usually occurs due to improper management and excessive fertilizer use. All plants were positioned and pruned in an open area and spaced 4 m apart within rows at 6 m intervals, with crops in between them. In addition, for green leaves, two-month-old self-rooted ‘Duli’ plants were sprouted from the seeds obtained from the fruit tree teaching practice base of Anhui Agricultural University. All seeds were sand-stored in a refrigerator (4℃) for at least two months before being planted in pots filled with steam‐sterilized soil and raised in a growth chamber until further use.

**Fig. 11:**
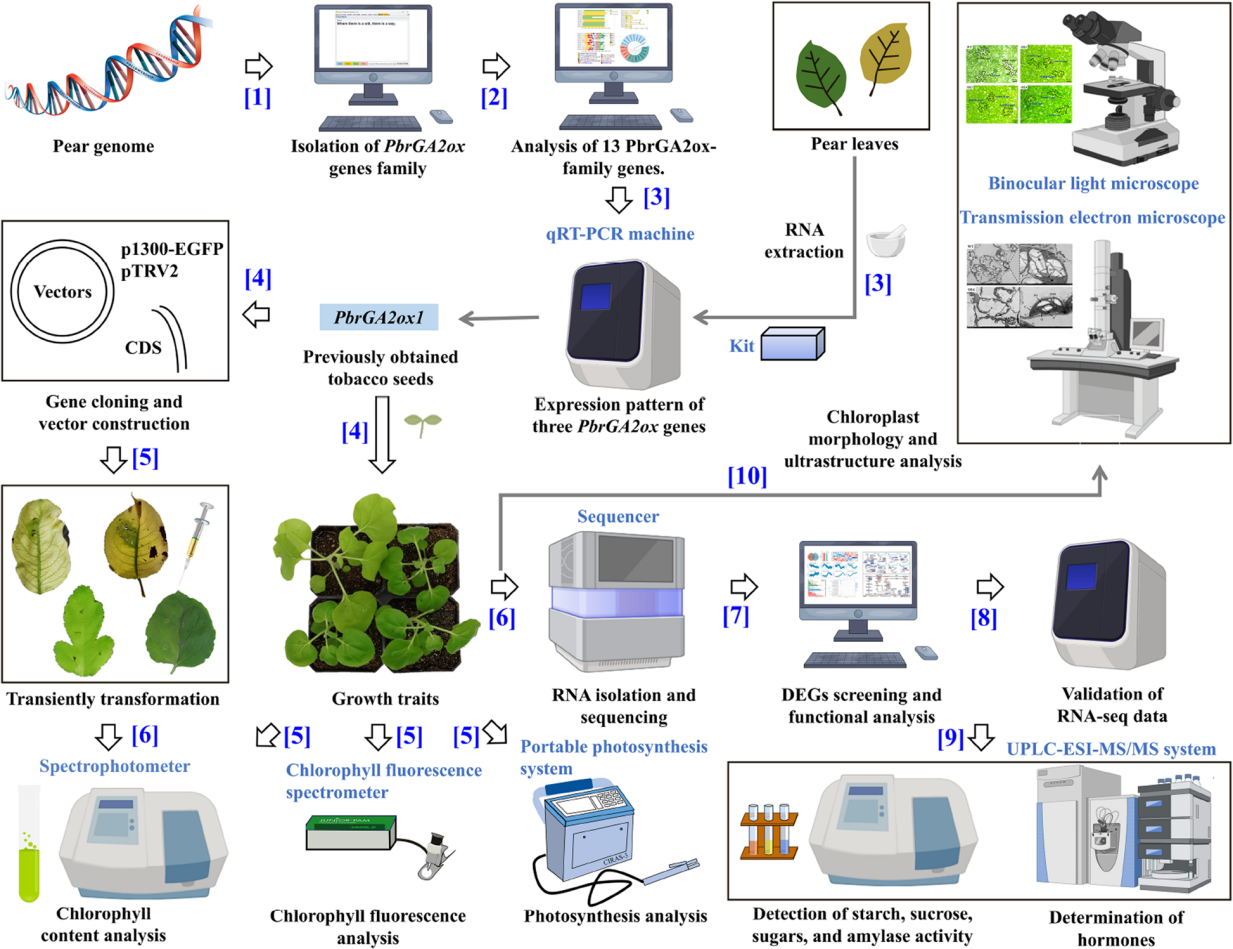
Flowchart of the summarized materials and methods introduced in this paper. The pictures were downloaded from the BioRender (https://www.biorender.com/) and Pinclipart online websites (https://www.pinclipart.com/). The numbers alongside the arrows indicate the order in which the experiments were carried out

Tobacco (*N. benthamiana*), obtained from the School of Horticulture, Anhui Agricultural University, Hefei City, Anhui Province, China, was used as the WT tobacco in this study. Previously obtained *PbrGA2ox1*-overexpressing (OE) plants in the WT background [31] were introduced in this study. The seeds from these plants were sown in pots containing steam‐sterilized soil (nutrient soil: vermiculite = 1:1, v/v) and were vernalized for 2 d at 4℃ in the darkness. The pots were then placed in a growth chamber under long-day conditions with a photoperiod of 16 h light/8 h dark (25℃ ± 1℃) under a relative humidity of 80%. Here, white fluorescence (12,000 lx light intensity) light was used. The dynamic growth traits were monitored at 7, 21, 35, and 49 days after vernalization (DAV). The Chl content was analyzed at each recorded point, and the photosynthetic parameters and Chl fluorescence parameters were analyzed at 35 DAV (Fig. 11). The leaf samples extracted at the indicated time points were immediately frozen in liquid nitrogen after collection and stored at − 80℃ for further analysis.

### Identification and fundamental analysis of PbrGA2oxs in pear

The sequences of nine reported Arabidopsis (*Arabidopsis thaliana*) GA2ox proteins (GA2oxs) [9] were accessed from TAIR (https://www.arabidopsis.org/). The sequences were selected as the seed sequences (Table S1 in Additional File 2) to obtain the orthologs against the Chines white pear database (http://peargenome.njau.edu.cn/) using the TBtools software with a threshold of E-value ≤ 1e^−5^. The structural domains and motifs of the putative proteins were confirmed using the Batch CD-Search (https://www.ncbi.nlm.nih.gov/Structure/bwrpsb/bwrpsb.cgi) and MEME tools (https://meme-suite.org/meme/tools/meme), respectively. Based on the analytical results, PbrGA2oxs were ultimately isolated by eliminating the proteins with incomplete or duplicated sequences. Finally, a neighbor-likelihood (NL) phylogenetic tree was constructed in MEGA 7.0 software with bootstrap replications set to 1000 among the identified PbrGA2oxs with the nine AtGA2oxs. TBtools software was applied to visualize the conserved domains, motifs, and structures of *PbrGA2oxs* (Fig. 11). To identify the candidate *PbrGA2oxs* that might be involved in Chl accumulation, another NL phylogenetic tree was built using the identified PbrGA2oxs and other GA2oxs that are known to be involved in Chl accumulation. The tree was visualized using either MS PowerPoint 2016 or the EvolView tool (https://www.evolgenius.info/evolview-v2/).

### Vector construction and transiently transformation

To induce transient overexpression in pear leaves, the full-length *PbrGA2ox1* gene without the stop codon was amplified using the primer pair *PbrGA2ox1*-EGFP-F/R (Table S5 in Additional File 2) and framed into the p1300-EGFP vector digested at XbaI and BamHI sites to generate overexpression fusion constructs *PbrGA2ox1*_OE with upstream CaMV 35S promoter, termed as 35S: *PbrGA2ox1* in our previous work [30]. Then, the *PbrGA2ox1*_OE fusion vector was transformed into the *Agrobacterium tumefaciens* GV3101-pSoup cells via thermal stimulation*.* The cells carrying either the fusion vector or the empty vector (EV) were grown on Luria–Bertani (LB) liquid medium at 28℃ until OD_600_ = 0.9. Then the cells were collected and resuspended at OD_600_ = 0.9 in infiltration buffer containing 10 mM MgCl_2_, 10 mM 4-morpholineethanesulfonic acid, and 0.2 mM acetosyringone, and placed in the dark for 2 − 3 h without shaking [105]. Next, the cell culture was infiltrated into the leaves of ‘Dangshansuli’ and ‘Akizuki’ pear plants using needle syringes. The infiltration experiments were conducted at nightfall to facilitate strain infection.

For virus-induced *PbrGA2ox1* silencing in pear and tobacco leaves, tobacco rattle virus (TRV) was introduced, where an expression vector pTRV2 and an auxiliary vector pTRV1 must be used in conjunction. Briefly, approximately 400 bp conserved fragment of the target gene was cloned using *PbrGA2ox1*-TRV-F/R primer pair (Table S5 in Additional File 2) and inserted into the pTRV2 vector digested at BamHI and XbaI sites to produce the silencing fusion vector *PbrGA2ox1*_TRV2, along with the strong 35S promoter upstream (Fig. 11). The corresponding infiltration buffers carrying *PbrGA2ox1*_TRV2 vector, empty vector pTRV2 (TRV2), and pTRV1 were obtained as described previously for 35S: *PbrGA2ox1*. pTRV1 was incubated with an equal volume of either *PbrGA2ox1*_TRV2 or TRV2, followed by incubation in the dark for 2 − 3 h. Then, the cultures were separately infiltrated into 40-day-old ‘Duli’ pear green leaves and 35-day-old tobacco leaf epidermis using needle syringes [105, 106]. After undergoing *Agro*-infiltration for approximately 14 d, all the samples were collected and photographed using a camera at the end point time. Subsequently, the Chl contents were detected. At least two biological replicates were prepared and investigated for each treatment.

### Determination of Chl, photosynthetic parameters, and Chl fluorescence parameters

The Chl levels of the leaves were qualitatively analyzed as described previously with some modifications [107]. A portable photosynthesis meter (CIRAS-3, PP Systems, Boston, USA) was used to compare the net photosynthetic rate (*Pn*), stomatal conductance (*Gs*), transpiration rate (*Tr*), intercellular CO_2_ concentration (*Ci*), and water use efficiency (WUE, WUE = *Pn*/*Tr*) of WT and OE plants at 35 DAV based on the method previously described by Tan et al. [108]. All measurements were conducted during 9:00 a.m.–11:00 a.m. on a sunny day. The operation parameters comprised relative humidity of 75%, a cuvette flow rate of 300 cc∙min^−1^, leaf temperature of 25 ± 1℃, leaf-to-air vapor pressure deficit (VPD) of 2.0 − 3.5 kPa, atmospheric CO_2_ concentration of 400 μM, and actinic light intensity of 1200 μmol∙m^−2^∙s^−1^ photosynthetic photon flux density (PPFD). A mixture of red (90%), blue (5%), and white (5%) LEDs were used as the light source in the leaf chamber (Fig. 11). All data were logged after test stabilization, and at least three measurements were recorded for each test.

Apart from these, a portable JUNIOR PAM device (WALZ, Effeltrich, Germany) was applied to determine the Chl fluorescence parameters. The fully expanded leaves of WT and OE plants at 35 DAV were selected and subjected to dark adaptation for 0.5 h before analysis (Fig. 11). The values of minimal fluorescence yield (*F*_*0*_), maximal fluorescence (*Fm*), maximal quantum yield of PSII (*Fv*/*Fm*), photochemical quantum yield of photosystem II [*Y*(*II*)], electron transport rate (*ETR*), photochemical quenching (*qp*), and quantum yield of regulated energy dissipation [*Y*(*NPQ*)] of the dark-adapted leaves were directly acquired after specific analysis using WinControl-3 software [109]. Three independent analyses were conducted for each plant.

### Detection of hormone levels

To find out the differences in hormones metabolism, the concentrations of indole-3-Acetic acid (IAA), 1-aminocyclopropane-1-carboxylic acid (ACC), BGAs (GA_1+3+4+7_), jasmonic acid (JA), jasmonic acid-isoleucine (JA-Ile), methyl jasmonate (MeJA), salicylic acid (SA), abscisic acid (ABA), N6-(delta2-Isopentenyl) adenine, and N6-(delta2-Isopentenyl) adenosine, were detected in the leaves of OE and WT tobacco seedlings at 35 DAV as described previously by Balcke et al. [110] (Fig. 11). In total, a 0.1 g sample was extracted as homogenate using precooled 50% acetonitrile (v/v) at 4 °C using a mixer (TD-20, HIPIE, China). The homogenate was centrifugated at a speed of 12000 rpm at 4 °C for 3 min. The supernatant was purified using a C18 reversed-phase, polymer-based, solid-phase extraction (RP-SPE) cartridge. Then, the cartridge was flushed with 1 mL of 30% acetonitrile (v/v), and the fraction was collected. The fraction was subsequently dehydrated under a gentle stream of nitrogen and dissolved in insert-equipped vials containing 200 μL of 30% acetonitrile (v/v) for UPLC-ESI–MS/MS assay. The operating conditions for the UPLC analysis were set as previously described by Balcke et al. [110]. All experiments were carried out at least thrice per sample.

### Quantification of starch, sucrose, reducing sugar, and amylase activities

Sugar and starch contents in plant leaves were determined as described previously [111] with some modifications. Namely, roughly 0.1 g dry tobacco leaf sample at 35 DAV was ground into a powder in the presence of liquid nitrogen. The powder was then mixed with 5 mL of 80% ethanol and homogenized at 200 rpm for 1 h. The homogenate was centrifuged at 12,000 rpm for 10 min at room temperature (RT).

To determine the reducing sugar content, 2 mL of the extracted supernatant was boiled dry and dissolved in distilled water. Then, the solution was mixed with 2 mL of 1% (m/v) 3,5-dinitrosalicylic acid. The absorbance of the solution was recorded at 540 nm using a spectrophotometer (TU-1810PC, PERSEE, Beijing, China). For evaluating soluble sugar content, the supernatant was mixed with an equal volume of chloroform. The centrifuged supernatant (50 μL) was boiled for 15 min with 4.95 mL of anthrone reagent (containing 72% H_2_SO_4_, 500 mg∙L^−1^ anthrone, 10 g∙L^−1^ thiourea). The absorbance of the reaction mixture was immediately read at 620 nm using a spectrophotometer.

For starch content examination, the precipitate was dextrinized with 2 mL distilled water at 100℃ for 15 min, and mixed with 2 mL of 9.2 M HClO_4_ after cooling at RT. Then, the fluid mixture was centrifuged at a speed of 8,000 rpm at RT for 10 min to obtain the supernatant (diluted to 25 mL). Finally, 2 mL of the diluted solution was used to quantify starch content using the method adopted for measuring soluble sugar content with a conversion coefficient of 0.9. Starch was visualized in situ using the method previously described by Li et al. [101], where 0.1 M iodine solution (10% potassium iodide + 5% iodine) was applied. The amylase activities were detected according to Zhang et al. [112]. Three biological replicates per plant were examined in each experiment.

Moreover, the resorcinol method was used to measure the sucrose content [111]. Briefly, the aforementioned supernatant in preparation stage was decolorized using approximately 0.1 mg activated carbon powder and transferred into a 10 mL volumetric flask. After that, 1 mL of the decolorized solution was concentrated to 100 µL at 100℃ and mixed with 0.1 mL of 30% (m/v) KOH and again boiled for 10 min. The mixture was then cooled and color-reacted using 3 mL of anthrone reagent at 40℃ for 15 min, the absorbance of the reaction buffer was immediately recorded at 620 nm. At least three biological replicates per plant were accessed. All aforementioned experiments could be seen in Fig. 11.

### RNA isolation and sequencing

Leaves from three biological replicates of normally grown WT and OE transgenic tobacco plants at 35 DAV were selected for total RNA isolation using the Trizol reagent kit (R4801-01, Magen, Guangdong, China) following the manufacturer’s instructions. The concentration and quality of the total RNA isolated from each sample were measured by Nanodrop2000 (Thermo, Walsham, USA) at OD_260_ and OD_280_ (OD_260_/_280_ = 1.9 − 2.1). The integrity of the RNA was then verified by RNase-free agarose-gel electrophoresis, and the RNA Integrity Number (RIN) was determined by Agilent5300 (Agilent, California, USA). A total of 1 μg qualified RNA per sample was employed to construct cDNA libraries and sequences. The raw reads generated on the Illumina system were first trimmed and quality controlled by removing the adapters, paired reads with ˃10% unknown nucleotides (N), and low-quality primary sequences with a quality rating of < 50% (Q-value < 20) using FASTQ preprocessor (https://github.com/OpenGene/fastp). The clean reads were then separately mapped to the reference genome (http://nadh.ice.mpg.de/NaDH/download/overview). The transcript expression level was statistically calculated and quantified as the number of fragments per kilobase of transcript per million mapped reads (FPKM). The differently expressed genes (DEGs) in comparison groups OE vs. WT were summarized according to the negative binomial distribution using the DESeq2 (http://bioconductor.org/packages/stats/bioc/DESeq2/) under the screening criteria of *p*-value < 0.05 and |Fold Change (FC, OE/WT)|≥ 1.5. The total number of DEGs and the upregulated and downregulated genes were subsequently counted (Fig. 11). The ChiPlot online tool (https://www.chiplot.online/) and the software Graph Prism v.7.0 were used to construct the expression heatmap of DEGs.

### Functional analysis of DEGs

The screened DEGs were annotated against the following public protein databases Diamond v0.8.37.99 (https://github.com/bbuchfink/diamond) and ID mapping tool (https://www.uniprot.org/help/id_mapping): Non-redundant (NR, https://www.ncbi.nlm.nih.gov/refseq/about/nonredundantproteins/), Pfam (http://pfam.xfam.org/), Swiss-Prot (http://web.expasy.org/docs/swiss-prot_guideline.html), and Clusters of Orthologous Groups of proteins (COG, http://www.ncbi.nlm.nih.gov/COG/). All annotated genes were subsequently subjected to functional enrichment analysis of Gene Ontology (GO, http://www.geneontology.org) terms, and the Kyoto Encyclopedia of Genes and Genomes (KEGG, http://www.genome.jp/kegg/) pathway enrichments, which were implemented using the GOATOOLS v1.2.4 (https://github.com/tanghaibao/GOatools) and the KOBAS v2.0 software (http://kobas.cbi.pku.edu.cn/home.do), respectively. The enriched functional terms and pathways for the tested DEGs were regarded as ones with P-adjust < 0.05. Additionally, the STEM software (https://www.cs.cmu.edu/~jernst/stem/) was used to cluster the expression profiles of the DEGs based on their normalized log_2_|FPKM_OE/WT_| values (Fig. 11).

### Gene expression analysis with quantitative real-time polymerase chain reaction (qRT-PCR)

The first-strand cDNAs were synthesized from the corresponding RNA using a fluorescence reverse transcription kit with genomic DNA (gDNA) remover (R323, Vazyme, Nanjing, China), and qRT-PCR was conducted on an ABI StepOne qRT-PCR detection system (Applied Biosystems, Foster City, CA, USA) to calculate the target gene expressions with three biological replicates (Fig. 11). *Arabidopsis* homologous genes in pear and tobacco plants were screened using the BioEdit v7.0.9 software. The sequences of the related genes were obtained against the genome of the Chinese white pear and the genome of *N. attenuate*. 14 tobacco genes were selected for qRT-PCR validation. Gene-specific primer pairs were designed using the Primer Premier 5.0 software. The primers are listed in Tables S4 and S5 in Additional File 2. Tobacco *actin* (*NbActin*) and pear *actin* (*PbrActin*) genes were used as the internal controls for the normalization of gene expression. The 2^−ΔΔCt^ method was used to calculate the relative gene expression [113].

### Chloroplast collection and transmission electron microscopy

To visualize the differences in chloroplast development of OE and WT plants, the chloroplasts in the fourth leaves from the top (area of each leaf ≈ 10 cm^2^) of WT and OE seedlings at 35 DAV were collected as described previously [114] with some modification (Fig. 11). The acquired fraction containing chloroplasts was subsequently resuspended in an equal volume of chloroplast storage buffer (330 mM sorbitol + 50 mM HEPES–KOH, pH 8), and photographed. The chloroplast ultrastructure was observed by following previous work [98] (Fig. 11). Briefly, the leaves of WT and OE plants at 35 DAV were excised and fixed in the TEM fixative under a vacuum. After ethanol dehydration, the samples were embedded in Spurr resin and were cut into ultrathin sections using an ultramicrotome (EMUC6, Leica, Wetzlar, Germany). Then, the prepared sections were examined, and the images were captured using a transmission electron microscope (H-7650, Hitachi, Tokyo, Japan). To this end, the number of chromoplasts per cell for WT and OE lines was compared using ImageJ, and the size of chromoplasts was manually counted. In addition, the cell morphology of OE and WT lines was investigated using a conventional inverted microscope, and the cell size was subsequently measured using the ImageJ software.

### Statistical analysis

Unless specifically addressed, all the data in the present study are represented as mean ± standard deviation (SD) of at least three independent replicates. Microsoft Excel software (Version, 2017) was used for drawing histograms. The significance of differences was evaluated using Graph Prism v7.0 by one-way analysis of variance (ANOVA) followed by Tukey’s test, as indicated by asterisks or ‘ns’ (no significance). The level of the bars’ significance was determined by *p* < 0.05.

### Supplementary Information

**Supplementary Material 1.**

**Supplementary Material 2.**

## Data Availability

The data that support the results of this study are available from the corresponding author upon reasonable request. The result of RNA-seq was archived in the National Center for Biotechnology Information (NCBI) BioProject under the accession number PRJNA1031366.
