# Supplementary material for: Genome-wide identification of GA2ox genes family and analysis of PbrGA2ox1-mediated enhanced chlorophyll accumulation by promoting chloroplast development in pear
Source: BMC Plant Biol. 2024 Mar 4;24:166. doi: 10.1186/s12870-024-04842-x (PMC10910807; doi:10.1186/s12870-024-04842-x)
Supplement: Supplementary file 1 — Supplementary Material 1. [file 12870_2024_4842_MOESM1_ESM.docx]

**Additional File 1**


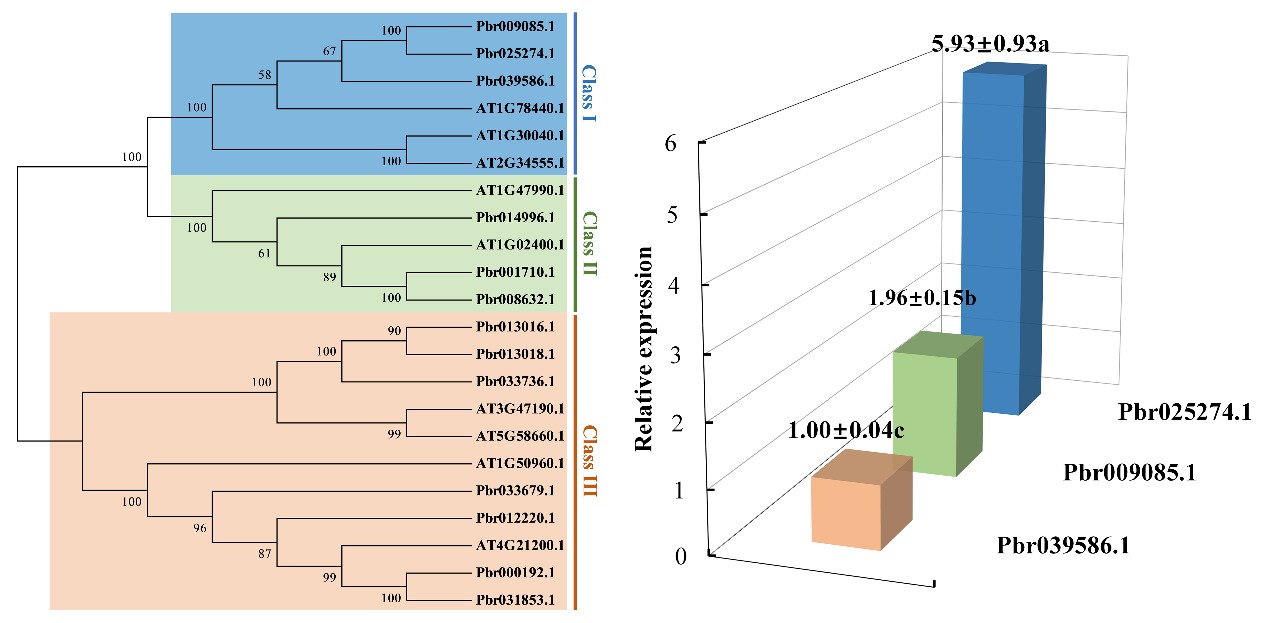


**(b)**

**(a)**

**Fig. S1** Isolation of candidate *PbrGA2ox* gene involved in chlorophyll accumulation. **(a)** Neighbor-likelihood (NL) phylogenetic tree of 13 PbrGA2ox proteins and nine AtGA2ox proteins. The tree was established using MEGA 7.0 software with 1,000 bootstrap replications, and the AtGA2ox proteins used here are AT1G02400.1, AT1G30040.1, AT1G47990.1, AT1G50960.1, AT1G78440.1, AT2G34555.1, AT3G47190.1, AT4G21200.1, and AT5G58660.1. **(b)** Expression profiles of *Pbr025274.1*, *Pbr039586.1*, and *Pbr009085.1* genes in normal pear leaves were analyzed using quantitative real-time PCR (qRT-PCR). The expression level of *Pbr009085.1* was used as the control and normalized to ‘1’. *PbrActin* was used as the internal reference gene. The values represent the mean ± SD of three biological replicates (n = 3). Different lower letters above the bars indicate a statistically significant difference at *p*＜0.05 (one-way ANOVA) to the control based on Tukey’s test, and the values alongside the letters mean the errors.


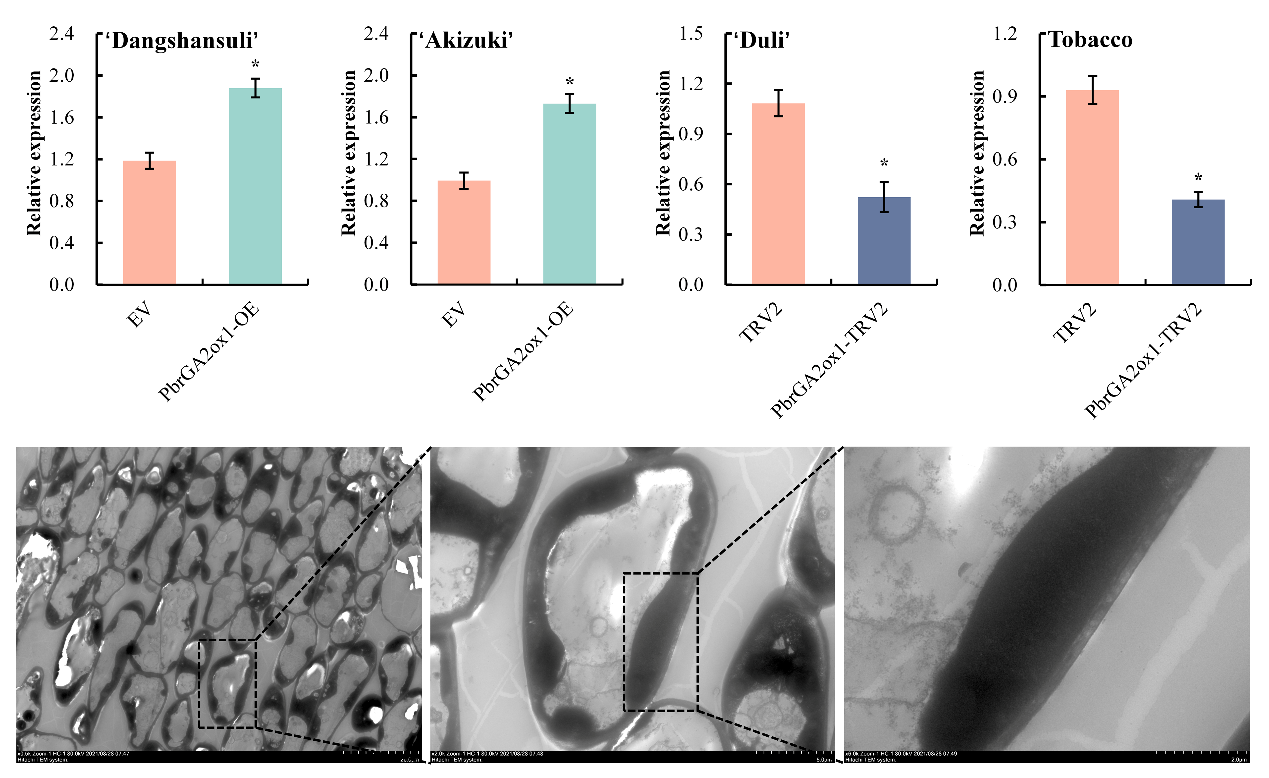


**(e)**

**(d)**

**(c)**

**(b)**

**(a)**

**Fig. S2** Analysis of the expression level of *PbrGA2ox1* in transiently infected leaves. **(a−b)** The expression level of *PbrGA2ox1* in transient overexpression system in leaves of ‘Dangshansuli’ and ‘Akizuki’ pear plants. **(c−d)** The expression level of *PbrGA2ox1* in transient silencing system in leaves of ‘Duli’ pear plants and tobacco plants, respectively. The injected sites were collected approximately seven days after infiltration of the corresponding strains, and quantitative real-time PCR (qRT-PCR) was conducted to detect the *PbrGA2ox1* transcript level. The data represent the mean ± SD (n = 3) of triplicates. The expression level calculated in leaves infected with the empty vector (EV) or the control vector TRV2 served as the control, *PbrActin* and *NbActin* were used as the internal reference genes. Asterisks used above the columns indicate significant differences at *p* < 0.05 (one-way ANOVA) to the control based on Tukey’s test. **(e)** The ultrastructure of chloroplasts obtained from the regreening spots of ‘Dangshansuli’ pear leaves after *PbGA2ox1* overexpression.

**
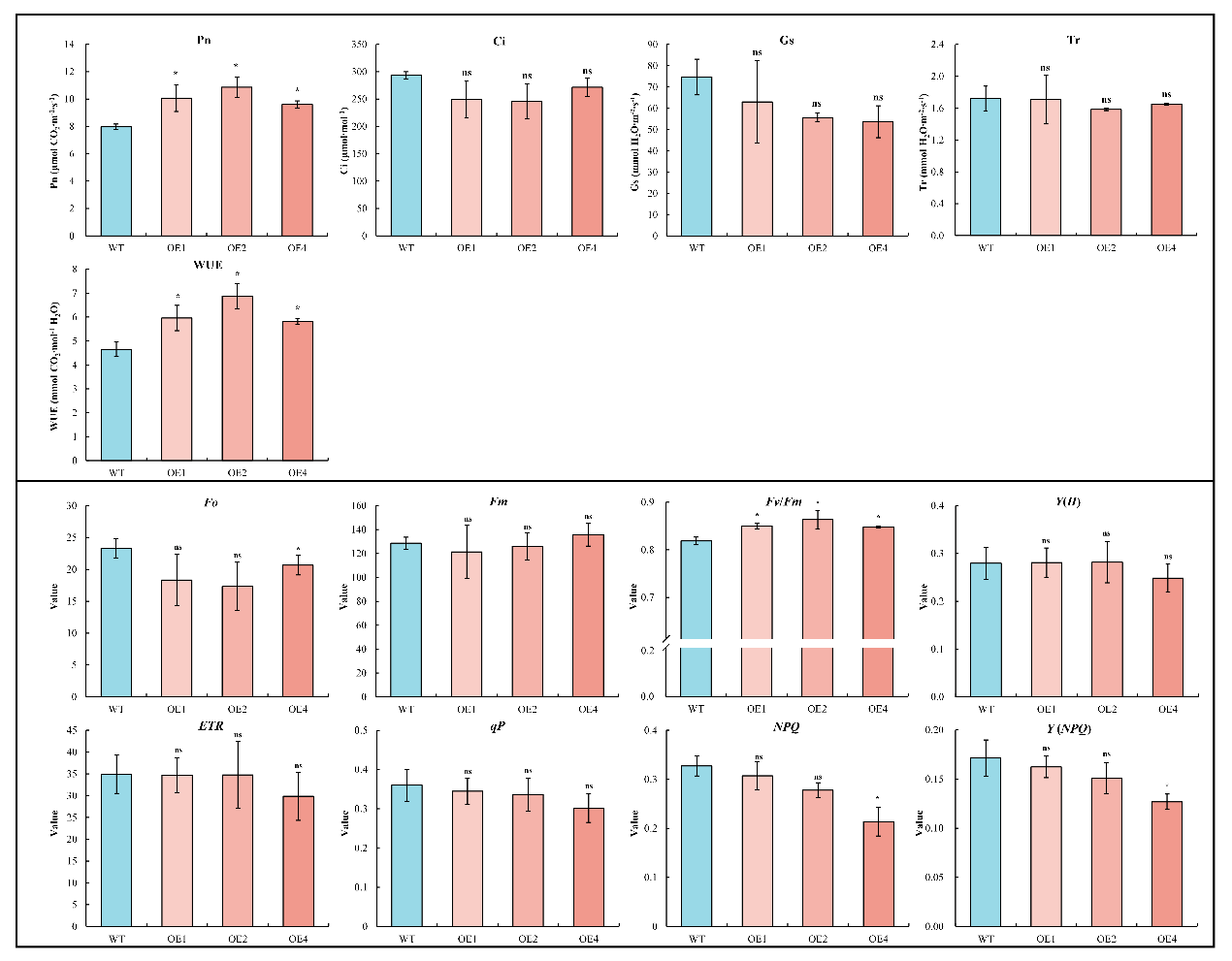
**

**(m)**

**(l)**

**(k)**

**(j)**

**(i)**

**(h)**

**(g)**

**(f)**

**(e)**

**(d)**

**(b)**

**(c)**

**(a)**

**Fig. S3** Comparative analysis of photosynthesis capacity and chlorophyll fluorescence for leaves of wild-type (WT) and *PbrGA2ox1*-overexpressing (OE) tobacco plants at 35 days after vernalization (DAV) under long-day conditions. **(a−e)** Photosynthesis parameters and **(f−m)** chlorophyll fluorescence parameters in the fourth fully expanded leaf from the shoot tip of WT and OE tobacco seedlings at 35 DAV under long-day conditions. The photosynthesis parameters are **(a)** the net photosynthetic rate (Pn), **(b)** intercellular CO_2_ concentration (Ci), **(c)** stomatal conductance (Gs), **(d)** transpiration rate (Tr), and **(e)** water use efficiency (WUE). These parameters are enclosed within red frames. The chlorophyll fluorescence parameters including **(f)** *F_0_*, **(g)** *Fm*, **(h)** *Fv*/*Fm*, **(i)** *Y*(*Ⅱ*), **(j)** *ETR*, **(k)** *qP*, **(l)** *NPQ*, and **(m)** *Y*(*NPQ*) are framed with green lines. The data represent the mean ± SD (n = 3) of three independent biological experiments. The indicator of WT serves as the control, ‘ns’ and asterisks used above the columns indicate insignificant and significant differences at *p* < 0.05 (one-way ANOVA) to the control based on Tukey’s test.


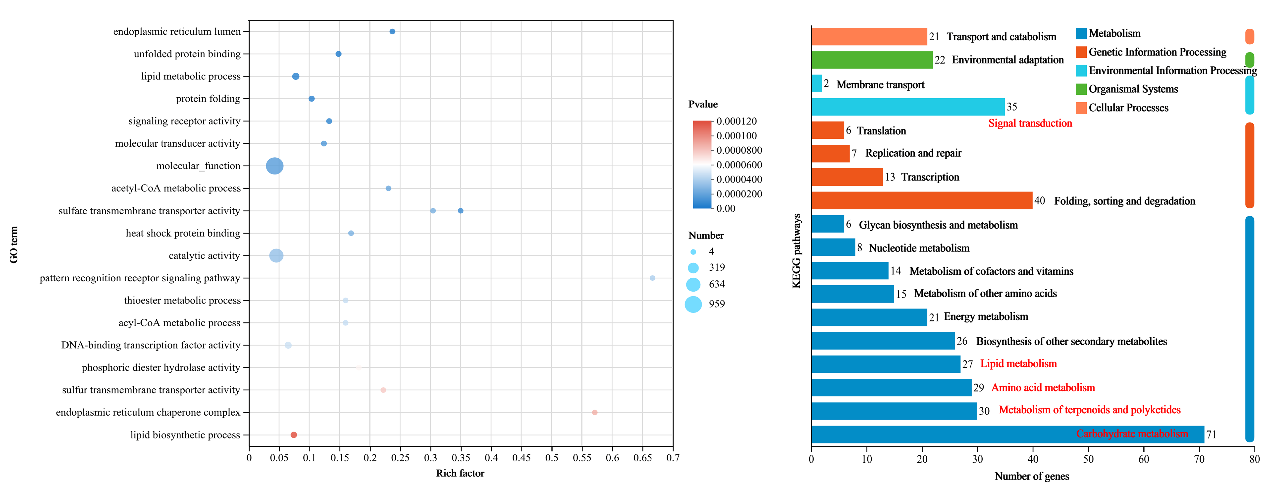


**(b)**

**(a)**

**Fig. S4** Functional enrichment analysis of D differentially expressed genes (DEGs). **(a)** A bubble map of the top 20 gene ontology (GO) enrichments for DEGs identified from the RNA sequencing (RNA-seq) data of the leaves from wild-type (WT) and *PbrGA2ox1*-overexpressing (OE) tobacco seedlings at 35 days after vernalization (DAV) under long-day conditions. The ordinate denotes the pathway name, the abscissa describes the richness factor, each circle in the map represents a pathway, the size of the circle indicates the number of DEGs in the pathway, and the color of these circles corresponds to different *p*-value ranges. **(b)** Enriched Kyoto Encyclopedia of Genes and Genomes (KEGG) pathways for DEGs. The pathways were classified into metabolisms, genetic information processing, environmental information processing, organismal systems, and cellular processes. The values indicate the number of genes in each classification, and the red marks highlight the most enriched category.


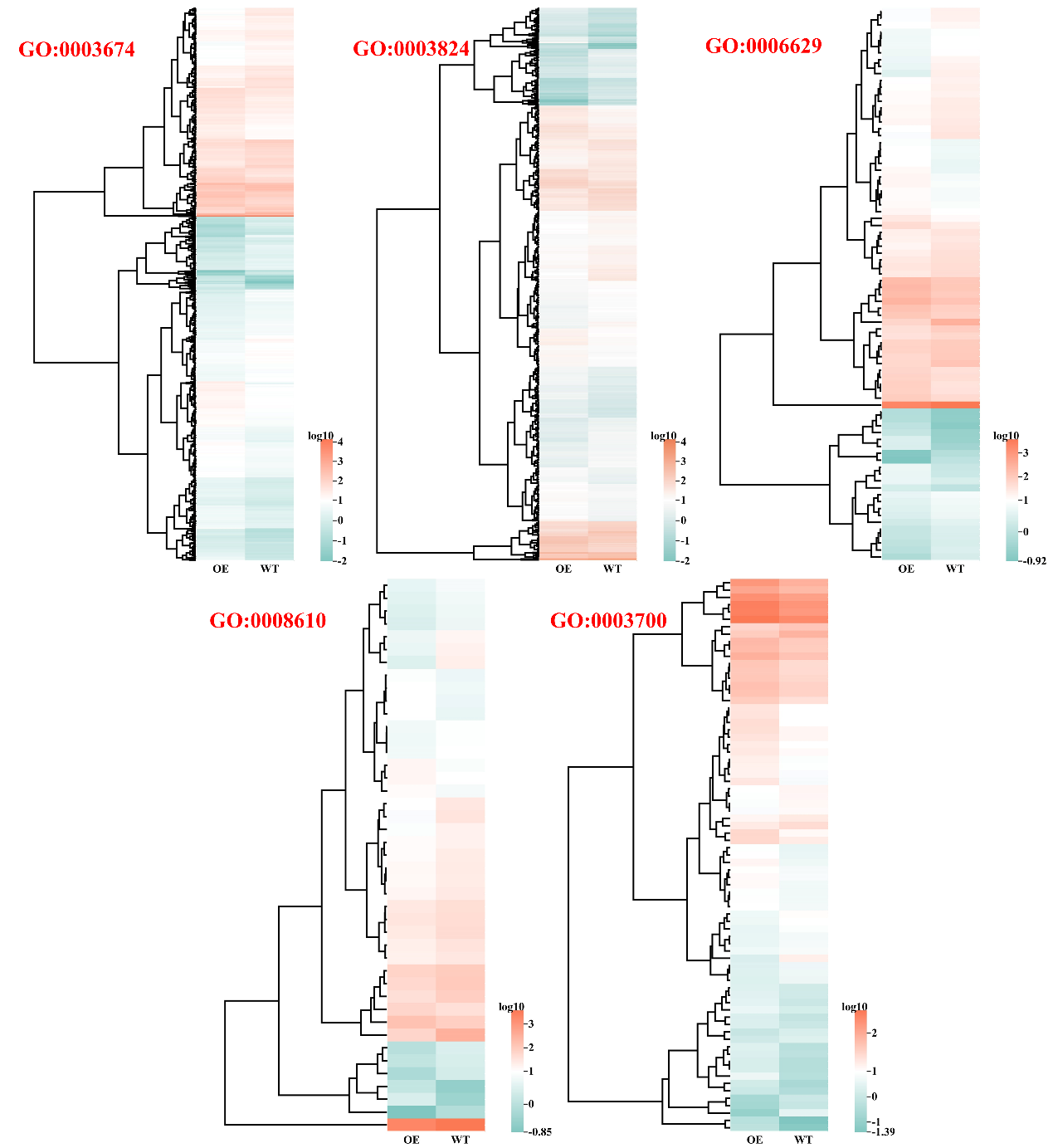


**(e)**

**(d)**

**(c)**

**(b)**

**(a)**

**Fig. S5** Expression heatmap of differentially expressed genes (DEGs) in several top enriched gene ontology (GO) terms. Heatmap visualization of DEGs in the **(a)** molecular function (GO:0003674), **(b)** catalytic activity (GO:0003824), **(c)** lipid metabolic process (GO:0006629), **(d)** lipid process (GO:0008610), and **(e)** DNA-binging transcription factor activity (GO:0003700) terms. Expression values were normalized to log_10_ counts based on the fragments per kilobase of transcript per million mapped reads (FPKM) values, and are presented in colors. Cyan to red gradient represents a gradual increase in gene expression.


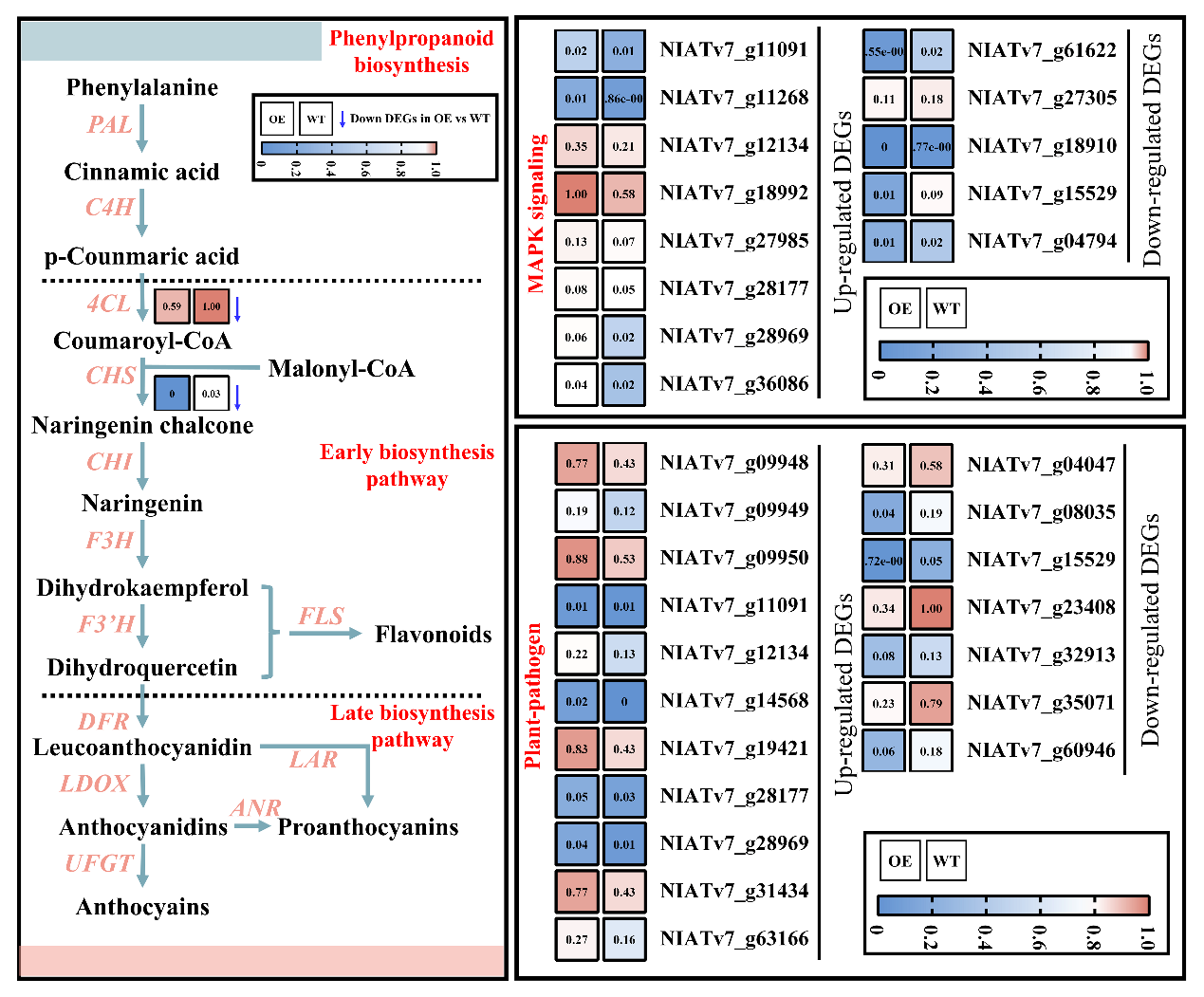


**(d)**

**(c)**

**(a)**

**(b)**

**Fig. S6** Pathways analysis of differentially expressed genes (DEGs) in leaves of wild-type (WT) and *PbrGA2ox1*-overexpressing (OE) tobacco seedlings at 35 days after vernalization (DAV) under long-day conditions. **(a−b)** Illustration of phenylpropanoid and flavonoid synthesis pathway in tobacco plants and the expression analysis of the related DEGs. Expression heatmap of DEGs involved in **(f)** MAPK signaling and **(g)** plant-pathogen interaction pathways. The expression levels were shown as min-max normalized counts based on the fragments per kilobase of transcript per million mapped reads (FPKM) value, and are presented in colors. Blue to red gradient represents a gradual increase in gene expression.

**
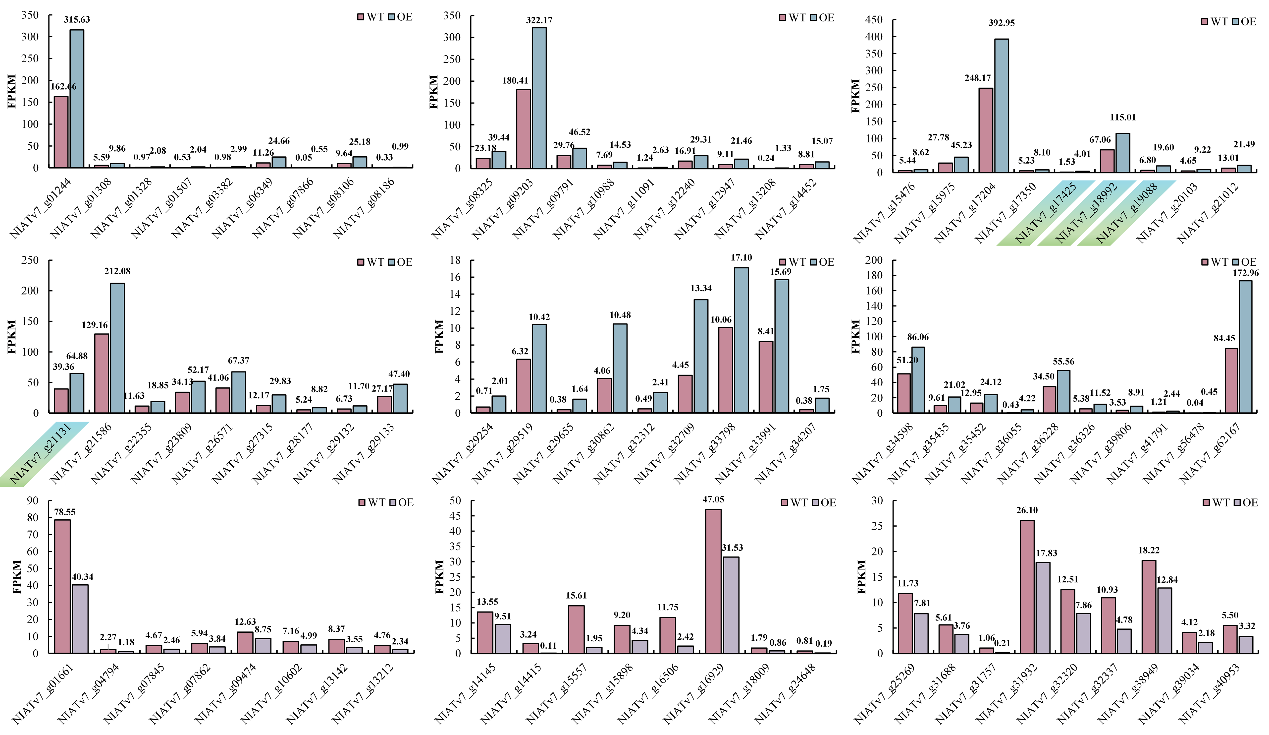
 Fig. S7** The expression profiles of the detected transcription factors among the differentially expressed genes (DEGs). The data represent the fragments per kilobase of transcript per million mapped reads (FPKM) values and are presented above the columns. Transcription factors involved in stress responses are denoted with blue-violet parallelograms.

**(c)**

**(b)**

**(a)**


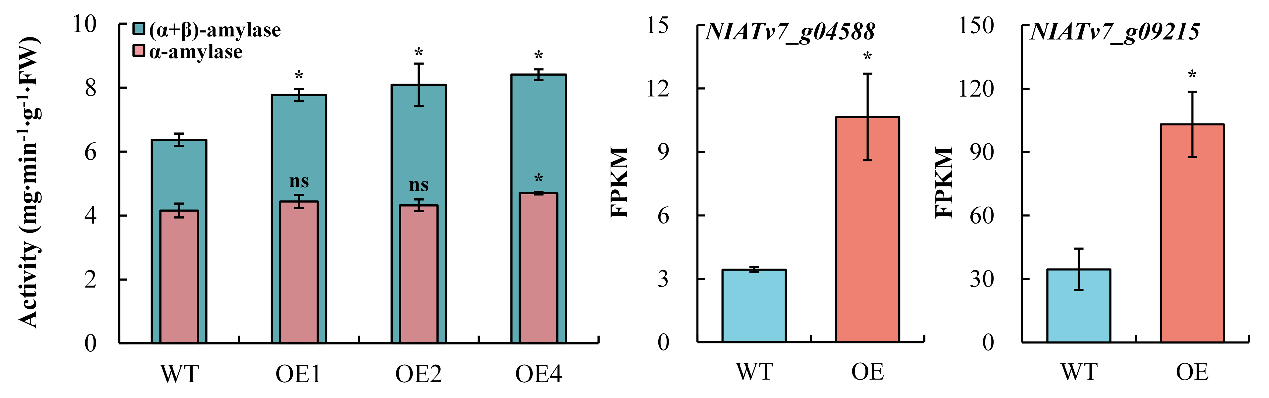


**Fig. S8** Activity analysis of the amylase (**a**) in leaves of wild-type (WT) and *PbrGA2ox1*-overexpressing (OE) tobacco seedlings at 35 days after vernalization (DAV) under long-day conditions. (**b−c**) The expression level of genes named *NbBAMs* which are responsible for the conversion of starch to sugar in tobacco plants. Values are presented in the form of log2 based on their FPKM obtained by RNA-seq, the values in WT serve as the control. ‘ns’ and asterisks used above the columns indicate insignificant and significant differences at p < 0.05 (one-way ANOVA) to the control based on Tukey’s test.

**
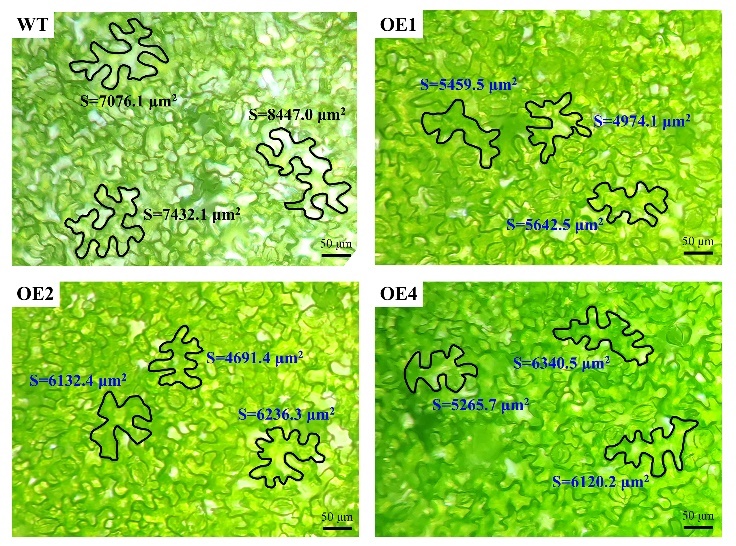
**

**Fig. S9** Cell morphology of leaves collected from wild-type (WT) and *PbrGA2ox1*-overexpressing (OE) tobacco seedlings at 35 days after vernalization (DAV) under long-day conditions. The outlines of the selected cells from WT and three OE lines (OE1, OE2, and OE4) were plotted with black solid lines. The values presented beside the cells represent the corresponding cell area calculated using the ImageJ tool. Scale bar = 50 μm.
