# Supplementary material for: Genome-wide identification of GA2ox genes family and analysis of PbrGA2ox1-mediated enhanced chlorophyll accumulation by promoting chloroplast development in pear
Source: BMC Plant Biol. 2024 Mar 4;24:166. doi: 10.1186/s12870-024-04842-x (PMC10910807; doi:10.1186/s12870-024-04842-x)
Supplement: Supplementary file 2 — Supplementary Material 2. [file 12870_2024_4842_MOESM2_ESM.docx]

**Additional File 2**

**Table S1** A list of GA2ox proteins used for the construction of a phylogenetic tree in this study

| **Gene ID** | **Accession number** | **Description** |
| --- | --- | --- |
| AtGA2ox1 | AT1G78440.1 | Arabidopsis thaliana gibberellin 2-oxidase 1 |
| AtGA2ox2 | AT1G30040.1 | Arabidopsis thaliana gibberellin 2-oxidase 2 |
| AtGA2ox3 | AT1G47990.1 | Arabidopsis thaliana gibberellin 2-oxidase 3 |
| AtGA2ox4 | AT1G50960.1 | Arabidopsis thaliana gibberellin 2-oxidase 4 |
| AtGA2ox5 | AT1G78440.1 | Arabidopsis thaliana gibberellin 2-oxidase 5 |
| AtGA2ox6 | AT2G34555.1 | Arabidopsis thaliana gibberellin 2-oxidase 6 |
| AtGA2ox7 | AT3G47190.1 | Arabidopsis thaliana gibberellin 2-oxidase 7 |
| AtGA2ox8 | AT4G21200.1 | Arabidopsis thaliana gibberellin 2-oxidase 8 |
| AtGA2ox9 | AT5G58660.1 | Arabidopsis thaliana gibberellin 2-oxidase 9 |
| PcGA2ox8 | AJ132438 | Phaseolus coccineus gibberellin 2-oxidase 8 |
| GhGA2ox1 | XP_016703412 | Gossypium hirsutum gibberellin 2-oxidase 1 |
| BnGA2ox2 | XP_013671430 | Brassica napus gibberellin 2-oxidase 2 |
| BnGA2ox6 | NP_001302845 | Brassica napus gibberellin 2-oxidase 6 |

**Table S2** Summary of sequencing statistics

| **Samples** | **Raw reads (G)** | **Clean reads (G)** | **Clean bases** | **Error rate (%)** | **Q20/Q30** | **GC content (%)** |
| --- | --- | --- | --- | --- | --- | --- |
| WT_1 | 4.24 | 4.21 | 6.41 | 0.0243 | 98.31/94.91 | 44.11 |
| WT_2 | 4.73 | 4.70 | 7.15 | 0.0246 | 98.19/94.59 | 43.39 |
| WT_3 | 4.43 | 4.40 | 6.69 | 0.0242 | 98.35/94.99 | 43.86 |
| OE_1 | 5.60 | 5.56 | 8.46 | 0.0245 | 98.22/94.68 | 43.82 |
| OE_2 | 5.50 | 5.46 | 8.30 | 0.0242 | 98.36/95.05 | 43.66 |
| OE_4 | 4.91 | 4.88 | 7.42 | 0.0244 | 98.26/94.75 | 43.35 |
| Total/mean | 29.41 | 29.21 | 44.43 | 0.0244 | 98.28/94.82 | 43.70 |

**Table S3** The top 10 up-regulated DEGs in OE vs. WT comparison with FPKM ≥ 1

| **Gene ID** | **Accession number** | **FC** | **Orthologs in *Arabidopsis*** | **Description** |
| --- | --- | --- | --- | --- |
| *DRN1* | *NIATv7_g27784* | 8.91 | *AT2G45180.1* | nsLTP family-related gene |
| *CAMAT2* | *NIATv7_g44291* | 8.74 | *AT5G64220.2* | Aluminum stress-related gene |
| *DJC26* | *NIATv7_g34402* | 8.53 | *AT4G13830.3* | DnaJ-like protein |
| *CYP78A5* | *NIATv7_g23299* | 8.13 | *AT1G13710.1* | Cytochrome P450 family |
| *AP180* | *NIATv7_g00102* | 7.44 | *AT1G05020.1* | ENTH/ANTH/VHS superfamily |
| *LBD10* | *NIATv7_g27916* | 6.97 | *AT2G23660.3* | LOB domain-containing protein |
| *ATJ3* | *NIATv7_g38092* | 6.50 | *AT3G44110.2* | HSP40 family |
| *XTH16* | *NIATv7_g34196* | 6.01 | *AT3G23730.1* | Xyloglucan endotransglucosylase/hydrolase |
| *ZFP1* | *NIATv7_g30202* | 5.94 | *AT1G80730.1* | Zinc finger protein |
| *ARR9* | *NIATv7_g26694* | 5.01 | *AT3G57040.2* | A response regulator |

**Table S4** The list of gene-specific primers used for qRT-PCR validation in this study

| **Gene ID** | **Accession number** | **Primer sequences**  **(F, 5'→3')** | **Primer sequences**  **(R, 5'→3')** | **Description** |
| --- | --- | --- | --- | --- |
| *NbActin* | *NIATv7_g33830* | TGGACTCTGGTGATGGTGTC | CCTCCAATCCAAACACTGTA | Actin 3 |
| *NbEIN3-like1* | *NIATv7_g18992* | AACATAAGCCGACAGGGGTG | TCTTCTTTCTGCGAGCCTGC | Ethylene insensitive 3-like 1 |
| *NbGH3.11* | *NIATv7_g23173* | TTGATCCGCCTGTCAGGAAC | TAGATCTGGTGGAGGTGGCT | Glycoside Hydrolase 3.11 |
| *NbTPS6* | *NIATv7_g07967* | TGGAAGTGATCAACCCGGAAG | TGCCTCGCGTAGTCAAAAGT | Trehalose-phosphate synthase 6 |
| *NbPΦB* | *NIATv7_g14936* | AGGTGCATGAGTATGAGTGGTG | AATACCTGCATGCCATCGCT | Phytochromobilin synthase |
| *Nb4CL* | *NIATv7_g17958* | CCGGAATCAATGTCAACGCC | CCGGAATCAATGTCAACGCC | 4-coumarate-CoAligase |
| *NbPDV1* | *NIATv7_g33437* | AGGATTTGCATGGCGAGGAT | TCTGAACCTCTTCGGCAAGG | Plastid division protein 1 |
| *NbMAPKK1-like* | *NIATv7_g12134* | GCTATGCAGTTGCGTTCCTC | CACCACCACCACATCGAACT | Mitogen-activated kinase kinase kinase 1-like |
| *NbAMY3* | *NIATv7_g09212* | CTTATGCACAAACCAAGCCATC | CCATTAAACTCACGTGCATCGC | Amylase 3 |
| *NbTPS7* | *NIATv7_g32888* | CTGCCACGGGTTATGACAGT | TCTGGCCTACGCTTAGCTTT | Trehalose-phosphate synthase 7 |
| *NbCHLM* | *NIATv7_g32557* | TCAACTATCCCACAATCATCGCT | TCTACTACTGCAACAGCGCC | Mg-chelatase enzyme |
| *NbCGF1B* | *NIATv7_g33515* | GTGCATCAAAAGCAGTGTGGT | CCTGCTAAGCACCTTGCACT | Chloroplast protein for growth  and fertility 1B |
| *NbSTK1* | *NIATv7_g27305* | GTGGAGTTATTTGTACAGGGAG | AAATTTATCAACTTGGGTGGGG | Serine threonine-kinase 1 |
| *NbCML19* | *NIATv7_g09948* | TCACTCAAAAAGGCAACAACA | GATAGCCTCACCGCCATCTC | Calmodulin like 1 |
| *NbHSK90.1-like* | *NIATv7_g19421* | TGCTCCTATGGCTTCAGTGC | AGCTACAGCAGAAGCCTCAC | Heat shock 90.1-like |

**Table S5** The list of gene-specific primers used for vector construction and qRT-PCR analysis in pear

| **Gene ID** | **Accession number** | **Primer sequences**  **(F, 5'→3')** | **Primer sequences**  **(R, 5'→3')** | **Description** |
| --- | --- | --- | --- | --- |
| *PbrGA2ox1* | *Pbr025274.1* | GAGCACACBGACCCACAAATC | GTGGTCCCCCAAAATAAATCAT | Pyrus x bretschneideri Gibberellin 2-oxidase 1 |
| *PbrGA2ox1-EGFP* | *Pbr025274.1* | AACACGGGGGAC**TCTAGA**ATGGTGTTTGTGACCAAATCAA | CCTTGCTCACCAT**GGATCC**TGCGGCTGCAATTCTCTC |  |
| *PbrGA2ox1-TRV2* | *Pbr025274.1* | GC**TCTAGA**GCAGATAAAACATATTGATAACAAGTAACAA | CGC**GGATCC**ATCATTCAAAGCAGAACAAAACTC |  |
| *PbrActin* | *Pbr024344.1* | TGGTGTCATGGTTGGTATGG | CAGGAGCAACACGAAGTTCA | Actin 7 |
| *PbrFtsZ1* | *Pbr042792.1* | TCCCTCAGAACGTGCATTCC | GCCGATTCCATGGGAGCAA | Filamenting temperature-sensitive Z proteins 1 |
| *PbrPDV1* | *Pbr037991.2* | TTTTCAACATCTGCGACGCC | GCAAAAGCAGAAGCCTCACG | Plastic division 1 |
| *PbrPIC1* | *Pbr026310.1* | GACTCGGCCCTTAGCGTATC | CCCTGATGGTTCCGATGGAG | Permease in chloroplast 1 |
| *PbrVAR2* | *Pbr022822.1* | CAAGGACTTTTGTGGCAGGC | CACTCCCCGCTCCAAGTAAA | Variegated |
| *PbrPDM4* | *Pbr013449.1* | ATCATCAAGGCCAAGGCCAA | GCGCTCTGACGTGCTTAATG | Pigment defective mutant 4 |
| *PbrARC5* | *Pbr021953.1* | GGCCTCTGGCACTGATTTGA | AGCAGTGCAGGCAACTTATCT | Accumulation and replication of chloroplast 5 |
| *PbrMCD1* | *Pbr015094.1* | TCGACTGAGAGCCATTGACG | CAATGCATAGACCGCAAGCC | Multiple chloroplast division site1 |
| *PbrCGF1* | *Pbr027408.1* | ACTCGCCCGTATTGAACTCC | CGCTGCTTAGAATCCCTGGT | Chloroplast protein for growth  and fertility 1 |
| *PbrCDP1* | *Pbr028282.1* | AGCTTATTGGCGTTCCTGCT | GAAGAAGGCAAAGTGCACCG | Chloroplast division site positioning 1 |

**Note: Bold and underlined letters represent the restriction enzyme site.**
